# Single-cell spatial proteomics maps human liver zonation patterns and their vulnerability to disruption in tissue architecture

**DOI:** 10.1038/s42255-026-01459-2

**Published:** 2026-02-20

**Authors:** Caroline A. M. Weiss, Lauryn A. Brown, Lucas Miranda, Paolo Pellizzoni, Sophia Steigerwald, Kirsten Remmert, Jonathan M. Hernandez, Karsten Borgwardt, David E. Kleiner, Shani Ben-Moshe, Florian A. Rosenberger, Natalie Porat-Shliom, Matthias Mann

**Affiliations:** 1 Proteomics and Signal Transduction, Max Planck Institute of Biochemistry, Martinsried, Germany; 2 Cell Biology and Imaging Sections, Thoracic and GI Malignancies Branch, Center for Cancer Research, National Cancer Institute, National Institutes of Health, Bethesda, MD USA; 3 Machine Learning and Systems Biology, Max Planck Institute of Biochemistry, Martinsried, Germany; 4 Surgical Oncology Program, National Cancer Institute, National Institutes of Health, Bethesda, MD USA; 5 Laboratory of Pathology, Center for Cancer Research, National Cancer Institute, National Institutes of Health, Bethesda, MD USA; 6 Department of Medical Biochemistry and Biophysics, Karolinska Institutet, Solna, Sweden

**Keywords:** Proteomics, Mechanisms of disease, Hepatocytes, Metabolism

## Abstract

Understanding protein distribution patterns across tissue architecture is crucial for deciphering organ function in health and disease. Here we show the application of single-cell Deep Visual Proteomics to perform spatially resolved proteome analysis of individual cells in native liver tissue. We built a robust framework comprising strategic cell selection and continuous protein gradient mapping, allowing the investigation of larger clinical cohorts. We generated a comprehensive spatial map of the human hepatic proteome by analysing hundreds of isolated hepatocytes from 18 individuals. Among the 2,500 proteins identified per cell, about half exhibited zonated expression patterns. Cross-species comparison with male mice revealed conserved metabolic functions and human-specific features of liver zonation. Analysis of samples with disrupted liver architecture demonstrated widespread loss of protein zonation, with pericentral proteins being particularly susceptible. Our study provides a comprehensive and open-access resource of human liver organization while establishing a broadly applicable framework for spatial proteomics analyses along tissue gradients.

## Abstract (web summary)

Using the single-cell Deep Visual Proteomics technique, the authors develop a resource providing spatially resolved proteomic analysis of individual cells in human liver tissue.

## Main

**Fig. 1:**
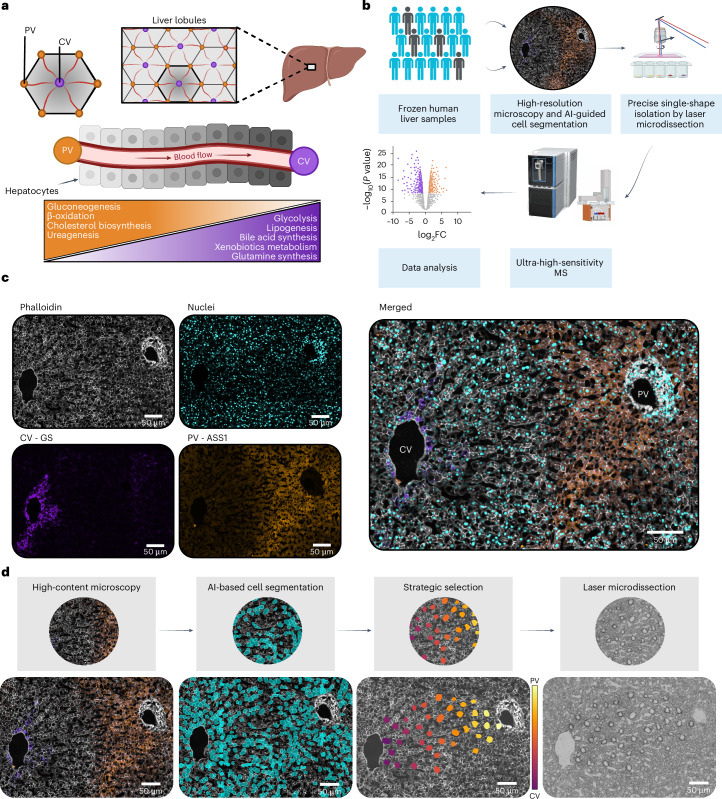
scDVP for spatially resolved proteomic analysis of liver zonation. **a**, Schematic representation of liver architecture showing the anatomical and functional organization of metabolic processes along the porto–central axis in human liver lobules. **b**, Workflow overview integrating high-resolution microscopy, artificial intelligence-guided cell segmentation, laser microdissection and ultra-high-sensitivity MS for spatial proteomic analysis. **c**, Immunofluorescence imaging of human liver tissue visualizing GS (pericentral; purple), ASS1 (periportal; orange), actin with phalloidin (cell boundaries; white) and nuclei with DAPI (cyan). **d**, Developed single-cell selection strategy of segmented contours (turquoise), strategic selection (colour gradient) and laser microdissection of equally distributed cells along a zonation trajectory. Representative image from one healthy individual. Immunofluorescence staining was performed once per individual (*N* = 18). AI, artificial intelligence; CV, central vein; FC, fold change; PV, portal vein. Illustrations in **a** and **b** created in BioRender. Weiss, C. https://biorender.com/q9yl3l9 (2026).

The liver, the largest internal organ, is crucial in orchestrating essential processes such as glucose homeostasis, protein synthesis, bile production and detoxifying harmful substances. To efficiently manage these diverse functions, the liver has evolved a unique anatomical organization. Directional blood flow through the hepatic lobule, the smallest functional unit, creates distinct metabolic and signalling gradients along this axis. Although histologically hepatocytes appear similar, they perform very different and often opposing metabolic functions depending on their location within the lobule—a phenomenon known as liver zonation^1–4^. For instance, periportal hepatocytes primarily engage in gluconeogenesis and fatty acid oxidation, while their pericentral counterparts specialize in glycolysis and lipogenesis^5^ (Fig. 1a). This sophisticated spatial division of labour allows the liver to perform competing metabolic processes simultaneously with remarkable efficiency. Liver pathologies commonly develop and progress in a zonated manner, with different regions showing distinct susceptibilities to various disease processes^3,6^. Therefore, precise mapping of liver zonation, with enhanced spatial resolution, is essential for a thorough investigation of liver physiology and disease. Accurately quantifying proteins as functional players is particularly important in liver disease, where translational and post-translational regulation, including protein turnover, often drives pathological processes^7–9^. While recent transcriptomic studies provided valuable insights into zonated expression patterns^10–12^, gene expression rarely corresponds to protein expression. A comprehensive understanding at the protein level is thus a vital first step for a better understanding of disease and the development of effective therapeutic strategies^13,14^.

Proteomic studies of liver zonation have so far relied on histological techniques and immunostaining of selected proteins, potentially biasing investigations towards already known directions. Mass spectrometry (MS)-based proteomics, on the other hand, allows an in-depth and unbiased analysis of the proteome within heterogeneous tissues^15^. We have recently introduced Deep Visual Proteomics (DVP), a technology that integrates high-resolution microscopy with artificial intelligence-guided image analysis, automated laser microdissection and ultra-sensitive MS^16^. DVP permits the analysis of collections of phenotypically similar cells from intact tissue while preserving spatial context. Recently, we enhanced the spatial resolution to examine the impact of the tissue environment on the proteome at the single-cell level^17^. The possibility of analysing protein expressions of single cells within their native tissue context revealed, in great detail, the spatial heterogeneity of metabolic functions along the portal to central veins in mice^17^. However, whether this spatial heterogeneity at the protein level is conserved in the human liver remains unknown.

Here we have substantially enhanced the technological framework of single-cell Deep Visual Proteomics (scDVP), enabling the precise quantification of protein gradients across hundreds of individual hepatocytes from a diverse cohort of 18 human liver samples. We developed an automated, strategic cell selection algorithm that streamlines the analysis of larger cohorts and maximizes information yield per tissue section. Furthermore, we established a robust statistical framework to quantify protein expression gradients along continuous spatial trajectories without introducing artificial binning. These advancements allowed us to comprehensively characterize human liver tissues, creating a spatial, single-cell proteomic map of protein gradients along the zonation axis. This map is openly accessible through our interactive web-based application (https://human-liver-dvp.streamlit.app/) providing user-friendly visualization of individual protein zonation profiles. We compared our findings in humans with our previously published mouse scDVP dataset to assess cross-species conservation. We further analysed samples displaying disrupted tissue architecture to examine how pathological perturbations affect spatial protein organization. Taken together, we established a robust analytical framework for spatial proteomics with broad applicability, even beyond liver research.

## Results

### Trajectory-driven cell selection enables scalable spatial proteomics in human liver

To create a comprehensive spatial map of the human hepatic proteome with single-cell resolution, we applied scDVP to a cohort of healthy and diseased human liver tissues across 14 healthy and 4 diseased individuals (Fig. 1b). Samples were obtained from individuals undergoing surgical procedures for either preventive (healthy cohort: *N* = 14; 11 female, 3 male) or curative (diseased cohort: *N* = 4; 1 female, 3 male) reasons. The cohort spanned a broad range of ages and body mass indices (Supplementary Table 1). Images of the liver sections were acquired using a four-channel immunofluorescence approach to gain critical spatial context: cell borders were stained by phalloidin and nuclei by DAPI, facilitating hepatocyte identification and segmentation. In addition, we stained samples with spatial markers to label pericentral (glutamine synthetase, GS) and periportal regions (argininosuccinate synthetase 1, ASS1) (Fig. 1c). These inversely zonated hepatocyte landmark proteins allow spatial navigation and hence enable identification of cell trajectories along the porto–central axis.

**Extended Data Fig. 1:**
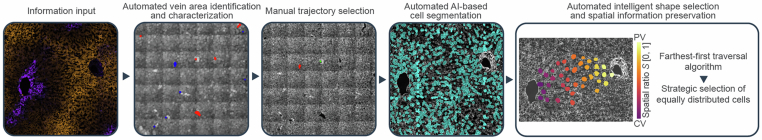
Automated selection of hepatocytes along zonation trajectories. The microscopy images are input for automated vein identification and characterization based on marker proteins. The user then manually selects suitable trajectories. Pre-segmented cell contours (turquois) are the basis for the farthest-first traversal algorithm, maximizing minimum distances between selected shapes to achieve uniform cell distribution along the portal-central axis. Selected shapes (colored dots) are assigned a spatial score *S* from 0 (CV, central vein) to 1 (PV, portal vein) based on their relative position.

**Extended Data Fig. 2:**
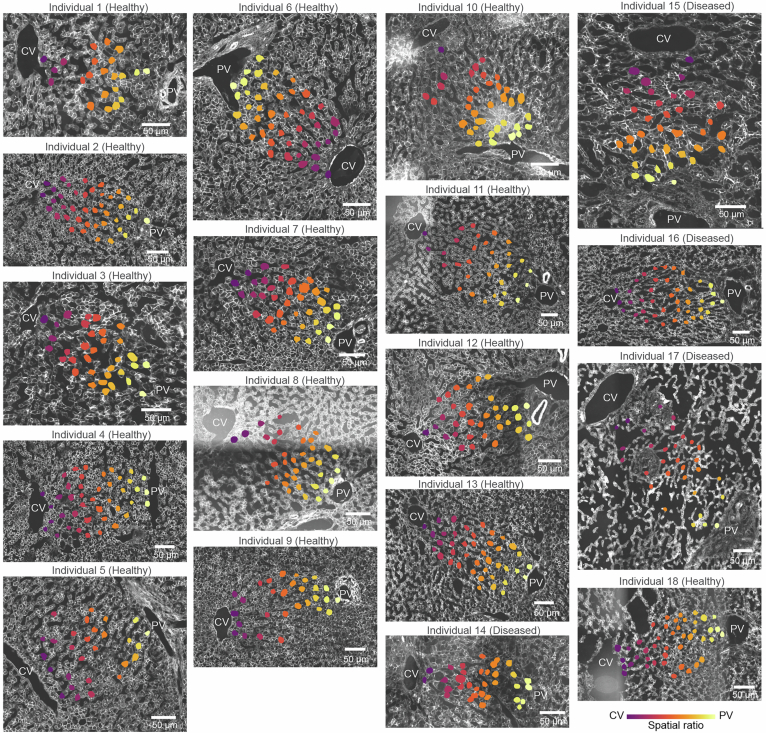
Overview of selected zonation trajectories across the human cohort. Representative microscopy images of liver sections from 18 individuals (healthy (*N* = 14) and diseased (*N* = 4)) highlighting hepatocytes along the chosen portal-central vein trajectories, which were subsequently isolated and measured by LC-MS (colored contours overlaid on phalloidin images). The color gradient indicates the spatial ratio *S* from a central vein (purple, *S* = 0) to the corresponding portal vein (yellow, *S* = 1). PV: portal vein, CV: central vein. Scale bars, 50 μm.

Our study presents a substantial increase in cohort size compared with our previous scDVP efforts investigating spatial trajectories, which examined up to three male mouse livers^17^. Another study scaled up scDVP to six individuals, but focused on direct spatial neighbourhoods rather than continuous trajectories^18^. To facilitate the increased scale, we aimed to maximize information yield gained from each tissue section while minimizing the cell number requirement. The large tissue sections obtained during surgery allowed the development of a framework for automated, strategic cell selection, enabling the analysis of a single zonation trajectory per sample. Our pipeline starts with automated identification and characterization of central and portal veins based on the marker proteins GS and ASS1, respectively. After manual selection of a clear zonation trajectory between identified veins, the algorithm employs a combinatorial optimization approach (called farthest-first traversal) to select segmented hepatocytes that are distributed uniformly along the porto–central axis^19,20^ (Methods). This approach maximizes the minimum distance between any two selected cells, ensuring comprehensive coverage of the zonation gradient while maintaining compatibility with downstream laser microdissection. Furthermore, comprehensive spatial metadata are recorded for each selected cell, including the critical spatial ratio parameter *S*—a normalized measure ranging from 0 (central vein) to 1 (portal vein) that quantifies the relative position along the zonation axis (Fig. 1d and Extended Data Fig. 1). In addition, we developed a graphical user interface (GUI) for manual definition of trajectory endpoints based on morphological features (Methods). This has proven to be essential when analysing samples with complex tissue architectures, for instance, in diseased states, where automated vein identification is challenging. We selected 44 cells per trajectory, providing approximately double coverage of the spatial domain spanned by ~20 hepatocytes across a human liver lobule and ensuring robust statistical analysis of zonation patterns (Extended Data Fig. 2). By analysing one optimal trajectory rather than dispersed cells, we capture the complete zonation gradient with fewer measurements while minimizing effects introduced by inter-lobule heterogeneity.

### Mapping the zonated proteome at over 2,500 proteins per single hepatocyte shape

**Extended Data Fig. 3:**
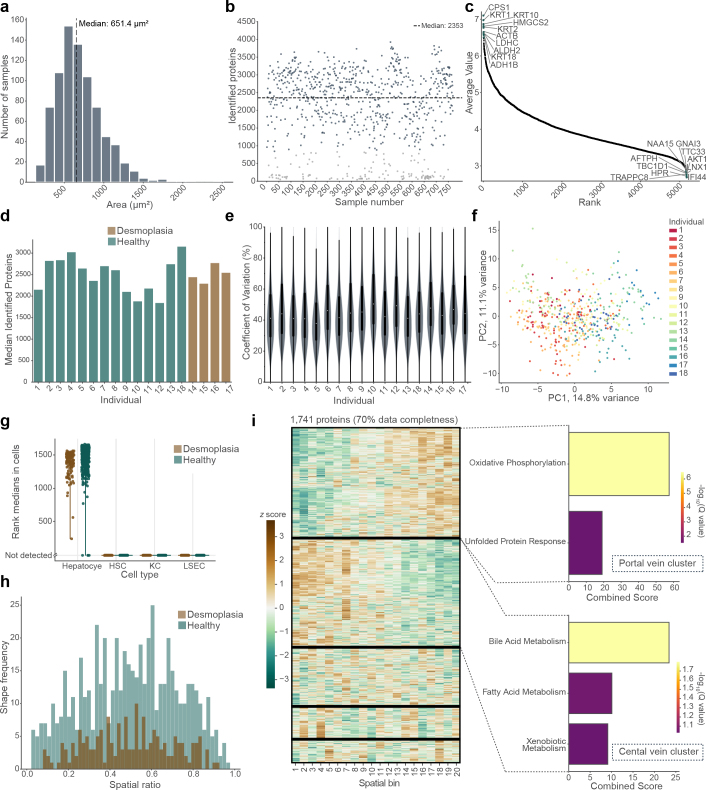
Data quality control of single-cell hepatic proteome dataset. **a-c**) Analysis of complete dataset before filtering (*n* = 792, *N* = 18): **a**) Histogram of all isolated hepatocyte shape areas. **b**) Number of identified proteins across all measured samples. The dotted line is the median number of quantified proteins. Grey points indicate samples with protein numbers below the threshold of 1.5 standard deviations from the median, which were excluded in later analysis steps (included = 649, excluded = 143). **c**) Protein abundance rank plot with the top and bottom 10 most abundant proteins highlighted. **d-f**) Analysis after standard deviation filtering per patient (*n* = 649, *N* = 18): **d**) Bar plots showing protein identification numbers across all individuals. The bars are colour coded in dependce to the association to the healthy (teal, *N* = 14) or the desmoplasia (brown, *N* = 4) cohort **e**) Violin and box plots showing coefficients of protein variation across all patients. Box plots inside violins show median (centre line), first and third quartiles (box bounds) and 1.5× interquartile range (whiskers). **f**) Principal component analysis of the entire cohort after outlier removal with samples colored by the patient (*n* = 513, *N* = 18). **g**) Distribution of median protein ranks for cell type-specific markers within individual isolated cells. Each dot represents one isolated cell shape (*n* = 441 shapes from healthy individuals, 118 shapes from desmoplasia patients). For each cell shape, the median rank was calculated across all 20 marker proteins for each cell type. High ranks indicate abundant proteins, while ranks of 0 indicate undetected proteins (stated as not detected). Cell types: hepatocyte, hepatic stellate cell (HSC), Kupffer cell (KC), liver sinusoidal endothelial cell (LSEC)). **h**) Histogram showing the frequency distribution of isolated shapes according to their spatial ratio *S* (0 = central vein, 1 = portal vein) in healthy (teal) and desmoplasia (brown) tissue samples. **i**) Protein expression heatmap from healthy individuals across spatial bins using proteins detected in at least 70% of samples (*n* = 1741, *N* = 14). Proteins were clustered using Ward’s hierarchical clustering with Euclidean distance. Bar plots show overrepresentation analysis of the two largest clusters results using MSigDB Hallmark collection with the complete dataset as background and statistical significance assessed using Benjamini-Hochberg correction for multiple testing (q < 0.1). The clusters were classified as portal and central in accordance with the terms.

We isolated a total of 792 single hepatocyte shapes with a median area of 651.4 μm^2^, corresponding to the expected cross-section of human hepatocytes in 10-μm tissue slices (Extended Data Fig. 3a). Given the average hepatocyte diameter of 20 to 30 µm and tissue sections of 10-µm thickness, microdissected samples corresponded to one-third to half a cell with approximately 250 pg of input material. For MS analysis, we employed the Orbitrap Astral mass spectrometer, which represents a substantial advancement in sensitivity compared with previous instrumentation^15^. We designed a sample-tailored data-independent acquisition (DIA) method which optimized the width of isolation windows based on the precursor density distribution of our samples (Supplementary Table 2). This allowed for efficient ion selection by employing narrower windows where precursor density is high and wider windows in sparse regions, maximizing peptide detection while minimizing interference. The resulting optimal coverage of existing precursors is especially critical when analysing ultra-low-input samples^21,22^.

**Fig. 2:**
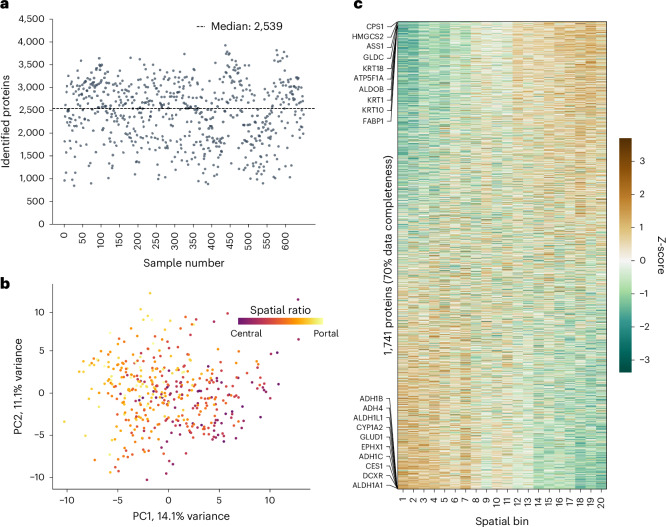
Deep proteome coverage enables spatial analysis of human liver zonation. **a**, Number of proteins identified per hepatocyte shape after quality filtering (*n* = 649, *N* = 18). The dotted line indicates the median depth across all included samples at 2,539 proteins. **b**, PCA of single-cell proteomes after outlier removal from healthy liver tissue. Each dot represents a single hepatocyte shape, with its colour indicating spatial ratio *S* (*n* = 413, *N* = 14). **c**, Protein expression heatmap (*Z*-scored) from healthy individuals across 20 equal-width spatial bins from central (spatial ratio *S* = 0) to portal (spatial ratio *S* = 1). Proteins are ordered by expression differences from portal (top) to central vein (bottom). The top ten proteins in each direction are labelled. Unless otherwise stated, only proteins detected in 70% of samples are included (*n* = 1,741, *N* = 14). PC1, principal component 1; PC2, principal component 2.

After applying quality threshold filtering, we identified a median of 2,539 proteins per shape with a maximum of 3,926 proteins (Fig. 2a and Extended Data Fig. 3b). We measured this study in a label-free manner and importantly at 80 samples per day (SPD; Methods), twice the throughput of previous scDVP studies. Total measuring time was only 10 days for this comprehensive single-cell proteomics study of 792 shapes. This identified 5,175 distinct proteins across the cohort, with the known hepatocyte markers CPS1 and HGMCS2 among the highest abundance proteins^23,24^ (Extended Data Fig. 3c). Of note, keratins were among the more abundant proteins, including those with documented hepatic expression, but without zonation patterns^23,25^ (Supplementary Fig. 1). The liquid chromatography–MS (LC-MS) workflow showed high robustness with a consistent median number of identified proteins, coefficients of variation and absence of pronounced per-person clustering in a principal component analysis (PCA; Extended Data Fig. 3d–f). There was no evidence of cross-contamination by other liver cell types (Extended Data Fig. 3g and Supplementary Fig. 2). Our strategic cell selection approach achieved uniform spatial distribution across zonation trajectories, with isolated shapes showing symmetric representation from central to portal vein regions (Extended Data Fig. 3h). Although hepatocyte size has been linked to zonal origin in three-dimensional measurements^26^, we found no correlation between shape area and spatial ratio (Spearman’s correlation = −0.042; Supplementary Fig. 3), likely reflecting the variable sectioning planes inherent to two-dimensional tissue analysis. Among samples from healthy individuals, we found a strong correlation between the position along the porto–central axis and the first component in PCA, indicating that space is the main driver of data variance (Spearman’s correlation = −0.55, *P* = 3 × 10^−34^, *n* = 413, *N* = 14; Fig. 2b).

Spatial analysis of the liver proteome has so far relied on discrete binning approaches, commonly using three zones along the zonation axis^3^. This was recently increased to 20 to minimize averaging of effects^17^. As a first step, we likewise sorted the hepatocytes into 20 equal-width bins along the trajectory according to their spatial ratio *S* and calculated the mean protein expression in each bin. Ordering proteins by their expression change clearly visualized the spatially dependent expression of the human liver proteome (liver zonation)^1–4^ (Fig. 2c), consistent with previous findings in mice^17^. Hierarchical clustering of the binned data identified distinct protein expression clusters. Overrepresentation analysis of the two largest clusters revealed functional pathways characteristic of portal and central regions, such as oxidative phosphorylation or bile acid metabolism, respectively (Extended Data Fig. 3i). Together, the analysis demonstrates that the single-cell dataset captures the human hepatic proteome with high spatial fidelity.

### Single-cell protein gradient mapping defines spatial-dependent functionality of hepatocytes

**Fig. 3:**
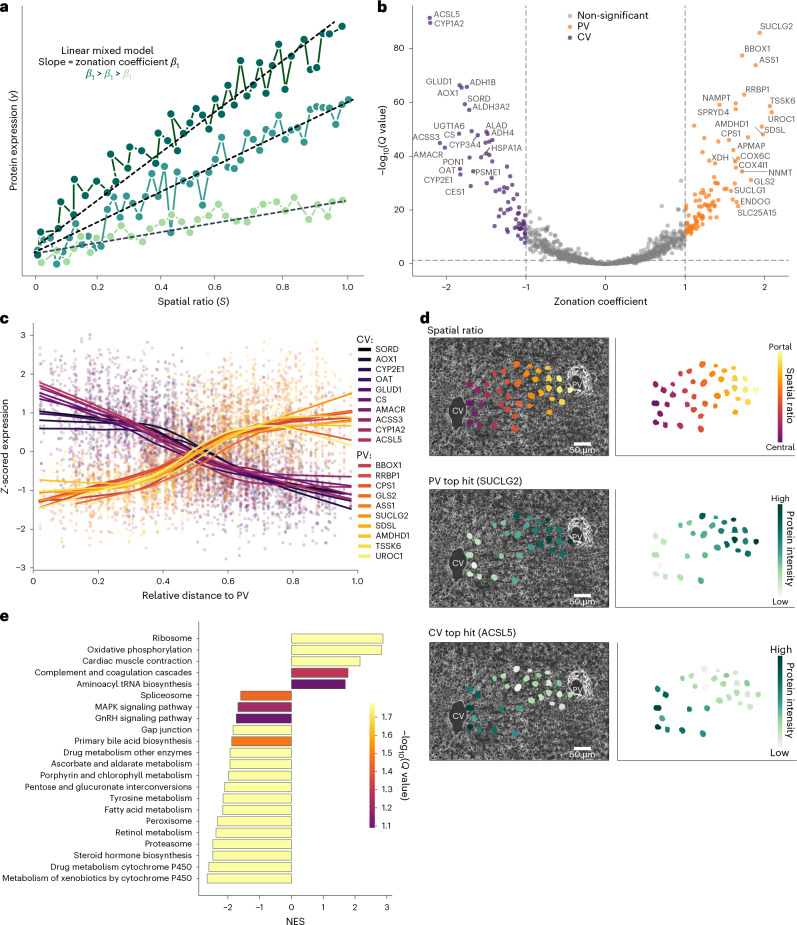
Gradient mapping of protein zonation patterns in healthy liver tissue. **a**, Schematic representation of continuous analysis approach based on an LMM to quantify protein zonation patterns. Each line represents a protein expression pattern across the porto–central axis after normalization, with steeper slopes (*β*_1_, zonation coefficient) indicating stronger zonation. The model accounts for patient-specific variations through random intercepts. **b**, Volcano plot showing results of the continuous analysis. Each protein zonation coefficient (*β*_1_) determined by LMM is plotted against the −log_10_(*Q* value) from a two-sided Wald test, which compares *β*_1_ against zero. *Q* values were calculated using Benjamini–Hochberg correction for multiple testing. The top 20 proteins from each side are labelled. Proteins with |zonation coefficient| > 1 and *Q* < 0.05 were defined as strongly zonated. **c**, Expression profiles of the top 20 most significantly zonated proteins (purple shades, central vein; orange shades, portal vein). Individual measurements are shown as dots overlaid with LOWESS smoothing curves (fraction = 0.4). **d**, Data visualization of protein zonation on microscopy image. Top: example trajectory with spatial ratio colour scheme. Middle and bottom: protein intensities (green, low to high) of the most significantly zonated proteins in portal (SUCLG2) and central (ACSL5) regions. **e**, GSEA ranked by zonation coefficient using the KEGG pathway database. NES were shown with statistical significance assessed using Benjamini–Hochberg correction for multiple testing (colour gradient, *Q* < 0.1). Negative NES indicated enrichment towards the central vein, and positive NES enrichment towards the portal vein. Unless stated otherwise, only proteins with 70% data completeness are included (*n* = 1,741, *N* = 14).

Isolating single shapes by laser microdissection permits very high spatial resolution. To leverage this aspect of scDVP and avoid reducing data content via binning, we developed a statistical framework that analyses protein gradients continuously across space. Our approach is based on a linear mixed model (LMM), accounting for individual-specific variations, and includes proteins with a data completeness of at least 70% (ref. ^27^; Methods). Using the spatial ratio *S* as an independent variable, we modelled protein expression values as the dependent variable. This determined the parameter *β*_1_, henceforth referred to as zonation coefficient, which characterizes and quantifies the protein expression gradient, that is, the rate at which the expression changes along the zonation trajectory (Fig. 3a). Furthermore, we calculated a statistical *Q* value by testing against the null hypothesis that gradients are zero.

Consistent with previous estimations^10,13,17,28^, 47% of the identified proteins show a significantly zonated expression pattern (*Q* < 0.05). The fact that nearly half of the proteome shows spatial organization underscores the remarkable diversity of hepatocytes at the single-cell level and emphasizes the importance of spatial context. By definition, negative coefficients of the spatial ratio *S* indicate proteins enriched towards the central vein, while positive zonation coefficients represent proteins with higher expression towards the portal vein. Their magnitude is a quantitative measure of zonation strength, enabling us to rank proteins by their degree of spatial organization. Stringent thresholding on absolute zonation coefficients larger than 1 identified 171 strongly zonated proteins (|zonation coefficient| > 1 and *Q* < 0.05), with 69 showing central vein enrichment and 102 portal vein enrichment (Fig. 3b). Among these were well-established periportal markers including SDSL, CPS1, HAL, PCK1 and C3, as well as pericentral markers such as CYP3A4, CYP2E1, OAT, GLUD1 and CYP1A2, further validating our approach. We provide comprehensive community access to our data through an interactive web-based application (https://human-liver-dvp.streamlit.app/) that enables dynamic exploration of individual protein zonation profiles derived from our continuous analysis. The data, including both the binned data structure and the continuous zonation coefficients, are also available in Supplementary Table 3.

**Extended Data Fig. 4:**
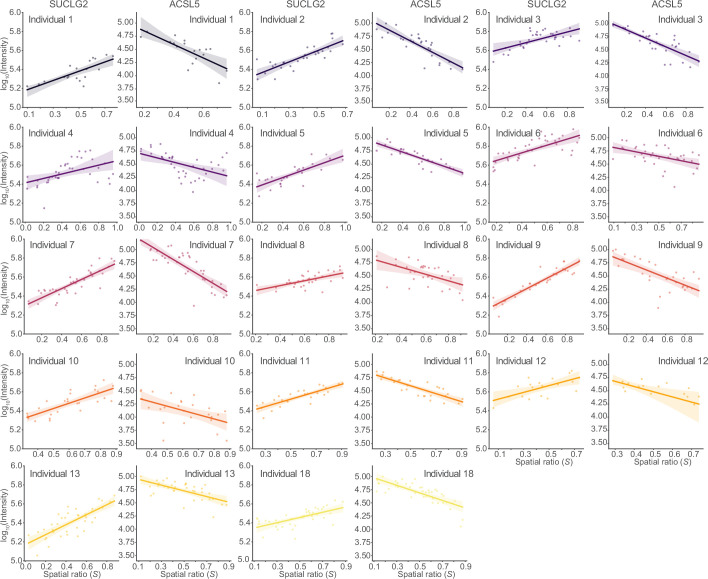
Individual patient analysis of the most significantly zonated proteins. Protein intensities (-log_10_ transformed) of the two most significantly zonated proteins (SUCLG2: periportal, ACSL5: pericentral) plotted against spatial ratio *S* per individual. Each dot represents a single hepatocyte measurement. Lines represent linear regression fits (centre) with 95% confidence intervals (shaded areas). Data include solely the healthy human cohort with proteins showing at least 70% data completeness (*n* = 1741, *N* = 14).

We visualized the top 20 significantly zonated proteins at the cohort level, revealing clear and continuous gradients along the porto–central axis (Fig. 3c). We showed for the two most significantly zonated proteins, SUCLG2 and ACSL5, that this trend was consistently present across individuals and further visualized the spatial dependency on the original microscopic image of one individual (Fig. 3d and Extended Data Fig. 4). This ability to map MS-based proteomics data back onto the original microscopy images illustrates the unique strength of DVP to connect molecular profiles with spatial tissue context, bridging the gap between imaging and functional data. Gene set enrichment analysis (GSEA) of proteins ranked by their zonation coefficient revealed distinct functional organization between portal and central regions in healthy liver tissue, indicated by positive and negative normalized enrichment scores (NES), respectively (Fig. 3e). Portal regions showed high enrichment of oxidative phosphorylation components. In contrast, central regions were characterized by elevated xenobiotic and steroid hormone metabolism. This spatial separation of key metabolic functions recapitulates known functional compartmentalization of the liver lobule, where periportal hepatocytes specialize in energy-intensive processes and pericentral hepatocytes in detoxification^3,4^.

**Extended Data Fig. 5:**
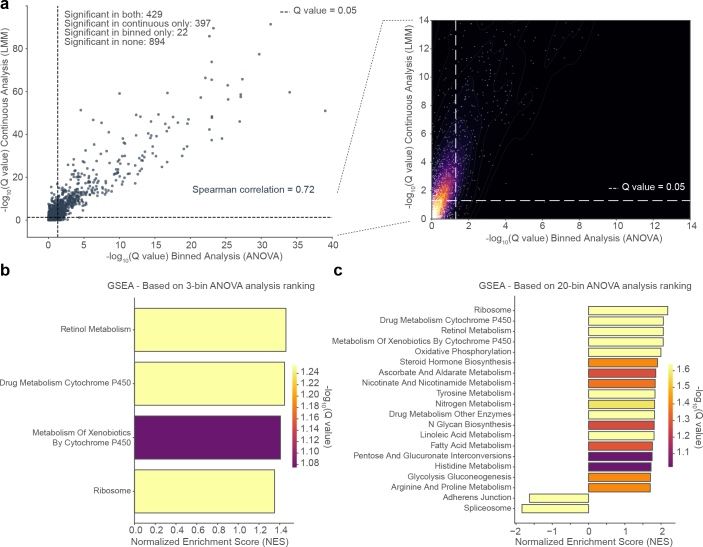
Comparison of classical binned vs novel continuous analysis approach. **a**) Left: Statistical significance comparison between continuous (LMM-based) and binned (ANOVA-based) analysis. For the binned analysis, protein intensities were divided into 20 equal-width spatial bins and analyzed using one-way ANOVA to test for expression differences between bins. Each dot represents a protein with its respective -log_10_(Q value). Numbers of proteins significant in both, either, or neither analysis and Spearman correlation coefficient are indicated. Right: Two-dimensional kernel density plot showing the distribution of -log_10_(Q value) with 20 density levels in the enlarged region (x: −3, 15; y: −8, 15). Individual proteins are shown as white scatter points overlaid on the density plot. Dashed lines indicate significance threshold (q < 0.05). **b**) Gene set enrichment analysis (GSEA) results from healthy human liver dataset using 3-bin ANOVA analysis (ranked by F statistic) using KEGG pathway database. Normalized enrichment scores (NES) are shown with statistical significance assessed using Benjamini-Hochberg correction for multiple testing (color gradient, q < 0.1). **c**) Analogue analysis to b using 20-bin ANOVA analysis representing higher-resolution binned approaches. Unless stated otherwise, only proteins with 70% data completeness are included (*n* = 1741, *N* = 14).

To evaluate the continuous analysis approach, we performed detailed comparisons with conventional binned methods at both protein and pathway levels. Compared with a 20-bin-based one-way analysis of variance (ANOVA), the continuous analysis approach yielded lower *Q* values for moderately zonated proteins, reflecting higher sensitivity to gradual changes. While both strategies show similar trends at higher *Q* values (429 proteins significant in both, Spearman’s correlation = 0.72), the continuous approach detected 397 additional statistically significant proteins (Extended Data Fig. 5a). At the pathway level, where multiple protein signals are aggregated, we expected the differences between methods to be less pronounced than at the individual protein level. To investigate this, we expanded our GSEA using both 3-bin and 20-bin approaches for comparison (Extended Data Fig. 5b,c). The 3-bin analysis captures only fundamental zonation patterns due to spatial averaging effects within the large bins, showing clear differences from our continuous method. The 20-bin analysis shows results similar to our continuous approach, which is expected since the 20 bins serve as a reasonable proxy for a continuous analysis; however, it does not provide differentiation of portal versus central vein direction. Taken together, our continuous approach provides key advantages for pathway analysis: directional information through positive and negative enrichment scores, single quantitative coefficients per protein and eliminating the need to pre-define bin numbers. This will be particularly valuable for analysing tissue gradients in the future where optimal bin numbers are unknown a priori.

### Cross-species comparison reveals conserved and unique features of liver zonation

**Fig. 4:**
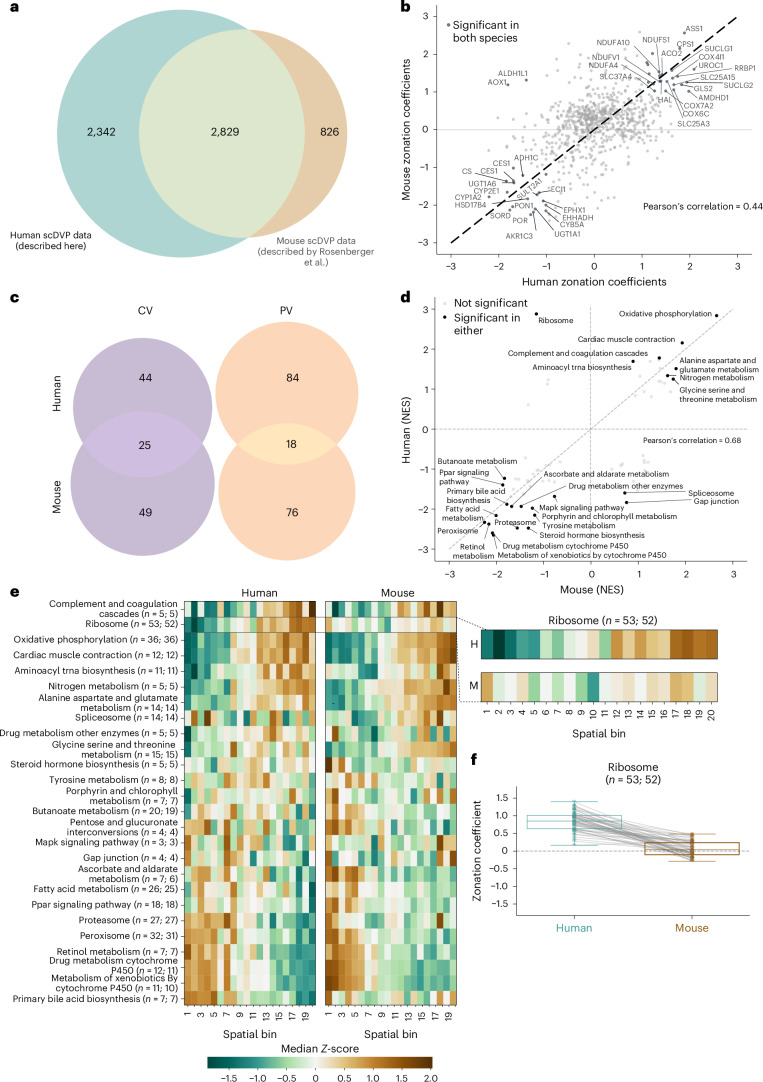
Cross-species comparison of liver zonation patterns between mouse and human. **a**, Overlap between proteins quantified in healthy humans (this study) and our previously published mouse scDVP dataset^17^ (human: *n* = 5,174, *N* = 14; mouse: *n* = 3,655, *N* = 3). **b**, Comparison of zonation coefficients determined by continuous analysis between humans and mice for shared proteins. Proteins with a |zonation coefficient| > 1 in both species are labelled. **c**, Comparison of differentially expressed portal and central vein proteins in mice and humans. **d**, Comparison of NES from independent GSEA (KEGG pathways) of human and mouse datasets, ranked by the zonation coefficient. Statistical significance was assessed using Benjamini–Hochberg correction for multiple testing and significantly enriched pathways (*Q* < 0.1) in either species are labelled. **e**, Heatmaps comparing expression patterns (*Z*-scored) of shared proteins across 20 equally spaced spatial bins from central (spatial ratio *S* = 0) to portal vein (spatial ratio *S* = 1) for significantly enriched pathways (*Q* < 0.1). The median *Z*-score of the proteins assigned to the respective pathway in each bin is displayed. The right panel shows a closeup of the ribosomal protein expression patterns in humans (H) and mice (M). The number of proteins in the respective term is indicated (H; M). **f**, Comparison of ribosomal protein zonation coefficients measured in human and mouse datasets (boxes show first and third quartiles, centre line indicates median, whiskers extend to 1.5× interquartile range). Individual proteins are shown as dots with connecting lines between matched proteins. Colours indicate human (teal) and mouse (brown) datasets. Unless stated otherwise, only proteins at 70% data completeness are included (human: *n* = 1,741, *N* = 14; mouse: *n* = 981, *N* = 3).

**Extended Data Fig. 6:**
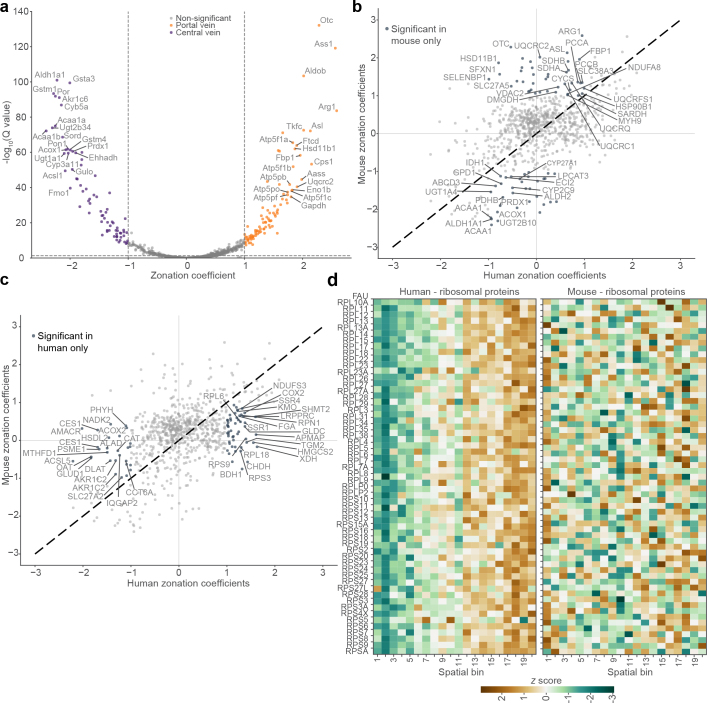
Cross-species analysis of liver zonation patterns. **a**) Application of continuous analysis to mouse scDVP data showing zonation coefficient β_1_ from linear mixed model plotted against -log_10_(Q value) after Wald test. Orange and purple dots indicate significantly enriched proteins (q < 0.05 and |coefficient | > 1) in the portal (PV; zonation coefficient > 1) and central vein (CV; zonation coefficient < −1) regions, respectively. **b**) Comparison of zonation coefficients between human and mouse shared proteins. Proteins with |coefficient | > 1 in mouse only are labeled and highlighted. **c**) Analysis as in b) highlighting proteins with |coefficient | > 1 in human liver tissue only. **d**) Protein expression heatmap (*z* scored) from detected proteins assigned to the KEGG ‘Ribosome’ term in humans (*n* = 88) and mice (*n* = 84). 20 equal-width spatial bins from central (spatial ratio *S* = 0) to portal (spatial ratio *S* = 1) are shown. Unless otherwise stated, only proteins at 70% data completeness are included (human: *n* = 1741, *N* = 14; mouse: *n* = 981, *N* = 3).

To investigate the extent of conservation of liver zonation between species, we compared the healthy human dataset (*N* = 14; 11 female, 3 male) with our previously published scDVP dataset acquired from male mice (*N* = 3)^17^. Despite some methodological differences between the two studies, including different MS platforms (Orbitrap Astral versus timsTOF SCP), sample preparation workflows (label-free versus dimethyl-labelled) and tissue types (surgically resected human versus perfused mouse liver), 2,829 homologous proteins were shared (Fig. 4a). To ensure consistency in the data analysis, we re-analysed the mouse data using the continuous gradient mapping framework. This identified 74 central and 94 portal vein-associated overlapping proteins at the stringent threshold filter (Extended Data Fig. 6a). Zonation coefficients in human and mouse datasets indicated an overall similar spatial distribution of protein expression profiles (Pearson’s correlation = 0.44; Fig. 4b). Twenty-five central and 18 portal vein-specific proteins maintained their strong zonated profile across both species (Fig. 4c). These evolutionarily conserved zonated proteins include key enzymes involved in critical liver functions such as urea cycle components (ASS1, CPS1), xenobiotic metabolism (CYP2E1, CYP1A2) and energy production (NDUFS1, SUCLG1), suggesting their fundamental importance for liver physiology across mammals.

In contrast, we also found that many proteins were zonated in one species but not the other, including two ambiguous homologous pairs (Extended Data Fig. 6b,c). We performed GSEA independently for each species for insights at the pathway level, ranking proteins by their zonation coefficients. The resulting NES values were highly similar between species, with a stronger correlation at the pathway level than at the individual protein level, reflecting greater conservation of functional modules than their individual components (Pearson’s correlation = 0.68; Fig. 4d). Visualizing protein zonation coefficients aggregated by functional pathways (*Q* < 0.1) revealed remarkably similar spatial patterns between mouse and human, exemplified by oxidative phosphorylation enrichment in the portal vein and enrichment of xenobiotic metabolism in the central vein (Fig. 4e). Interestingly, the zonation pattern of the abundant group of ribosomal proteins strikingly diverged between species (Supplementary Fig. 4). In humans, all detected ribosomal proteins consistently showed clear portal vein enrichment. This pattern was absent in mice at both pathway and individual protein levels (Fig. 4e,f and Extended Data Fig. 6d). This pattern was robust to alternative normalization strategies (core proteome normalization^17^) and was not observed for histone proteins, which served as non-zonated controls. Further, immunofluorescence staining for RPS3 recapitulated a subtle but consistent periportal trend (Supplementary Fig. 5). To our knowledge, the periportal enrichment of ribosomal proteins observed here has not yet been described in human liver zonation studies, while comparable mouse liver zonation studies have consistently shown no such enrichment pattern^28^. This finding may reflect species-specific variation in protein synthesis capacity across the liver lobule.

### Disruption of liver architecture shows asymmetric effects on protein zonation profiles

**Extended Data Fig. 7:**
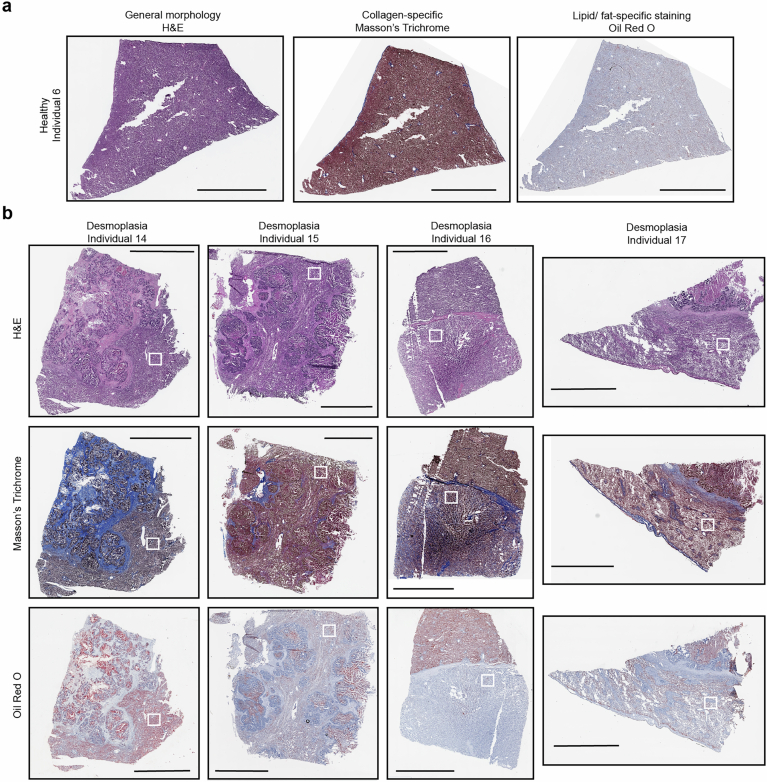
Histopathological assessment of surgical human liver tissue sections. **a**) Representative histological sections from a healthy individual (Individual 6). Left: Hematoxylin and eosin (H&E) staining for general morphology. Middle: Masson’s Trichrome staining for collagen-specific visualization. Right: Oil Red O staining for lipid/fat-specific visualization. **b**) Histological sections from all four individuals of the desmoplasia cohort (Individuals 14, 15, 16, and 17). Approximate location of analyzed zonation trajectories is illustrated by a white box in desmoplasia samples. Top row: H&E staining, middle row: Masson’s Trichrome staining, bottom row: Oil Red O staining. Stainings were performed once per individual. Scale bars, 5 mm.

Next, we investigated how liver disease affects spatial protein organization and expanded the analysis to individuals with metastatic liver disease. These surgical samples were obtained during resection procedures, providing large tissue specimens that enabled analysis of regions adjacent to but distinct from metastatic lesions. Histologically, the selected regions were characterized by desmoplasia, a clinical phenotype describing the formation of dense, fibrous connective tissue in response to invasive carcinoma (Extended Data Fig. 7 and Supplementary Table 4). Initial immunostaining of the zonation markers ASS1 and GS showed evidence of disrupted zonation profiles. This prompted us to develop the previously mentioned GUI for manual trajectory endpoint definition based on morphological features to enable the selection of cells even in this complex tissue environment. This critical extension permits scDVP application in samples that cannot be immunostained with traditional zonation markers.

**Fig. 5:**
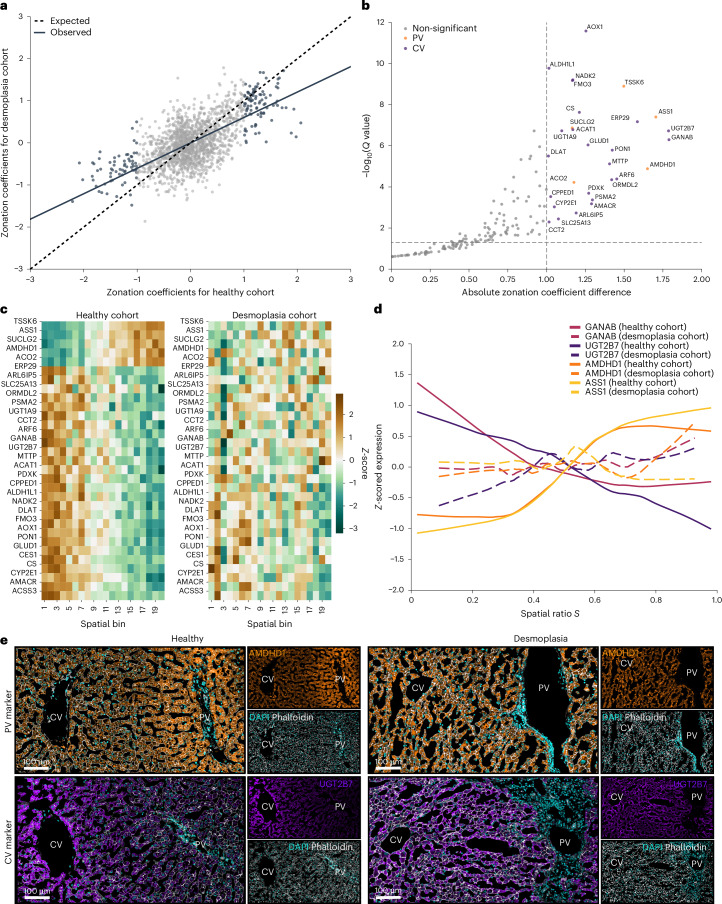
Loss of protein zonation patterns in human desmoplastic liver. **a**, Zonation coefficients in healthy and desmoplastic liver samples. Dotted line indicates theoretical unchanged zonation coefficients; solid line shows observed relationship as a linear regression line. **b**, Absolute differences in zonation coefficients between healthy and desmoplastic tissue. Only strongly zonated proteins (|zonation coefficient| > 1 and *Q* < 0.05) from healthy are analysed (*n* = 171). Proteins with significant zonation loss are highlighted and colour-coded by their original spatial enrichment. Their zonation coefficients were compared by Wald test (absolute zonation coefficient difference > 1 and *Q* < 0.05). **c**, Protein expression heatmap (*Z*-scored) from significant proteins in **b** in the healthy and desmoplasia cohorts. Twenty equal-width spatial bins from central (spatial ratio *S* = 0) to portal (spatial ratio *S* = 1) are shown. **d**, Expression profiles of the four proteins with the strongest absolute zonation coefficient difference. LOWESS smoothing curves (fraction = 0.4) are shown, with solid lines indicating the healthy and dotted lines the desmoplasia cohort. **e**, Immunofluorescence staining of portal vein (top; orange; AMDHD1)- and central vein (bottom; purple; UGT2B7)-associated protein in healthy (left; individual 12) and desmoplasia (right; individual 16) tissue. Phalloidin (white) and DAPI (cyan) label actin (cell boundaries) and nuclei, respectively. Unless otherwise stated, only proteins at 70% data completeness are included (healthy: *n* = 1,741, *N* = 14; desmoplasia: *n* = 1,684, *N* = 4). All panels within each row are at the same magnification.

**Extended Data Fig. 8:**
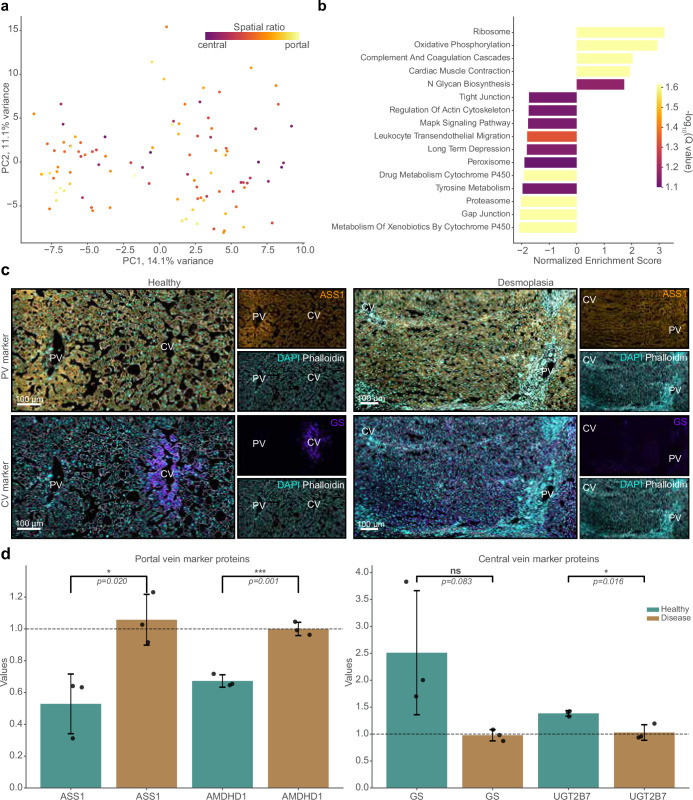
Loss of clear spatial architecture in desmoplasia liver tissue. **a**) Principal component analysis of single-cell proteomes from desmoplasia liver tissue. Each dot represents a single hepatocyte shape with color indicating its spatial ratio *S* (*n* = 100, *N* = 4). **b**) GSEA analysis ranked by zonation coefficient of the desmoplasia cohort using KEGG pathway database. Normalized enrichment scores are shown with statistical significance assessed using Benjamini-Hochberg correction for multiple testing (color gradient, q < 0.1). Negative NES indicates enrichment towards the central vein, and positive NES indicates enrichment towards the portal vein. Proteins were filtered to 70% data completeness are included (*n* = 1684, *N* = 4). **c**) Immunofluorescence staining of a portal vein (top; orange) and central vein (bottom; purple) associated protein in healthy (left; individual 4) versus desmoplasia (right; individual 16) tissue. Phalloidin (white) and DAPI (cyan) label actin (cell boundaries) and nuclei, respectively. PV: portal vein, CV: central vein. Scale bars, 100 μm. **d**) Normalized (CV/PV) average intensity showing the loss of zonal distribution in desmoplasia (*N* = 3) compared to healthy (*N* = 3). Two representative PV (ASS1 and AMDHD1) and CV (GS and UGT2B7) markers are shown. Data are presented as mean ± SD and individual data points are shown as dots. Statistical significance between conditions was assessed using two-sided independent t-tests. Significance levels: *** p < 0.001, ** p < 0.01, * p < 0.05, ns = not significant. Scale bars, 100 μm. All panels within each row are at the same magnification. Source data

To analyse spatial protein organization in desmoplastic tissue, we selected trajectories from regions adjacent to, but distinct from, metastatic lesions (Supplementary Fig. 6). Here hepatocyte morphology could be reliably assessed and the samples could be analysed orthogonal to their healthy counterparts (Extended Data Fig. 2). Notably, the number of proteins identified across both healthy and desmoplastic cohorts was consistent, indicating that proteome integrity was largely maintained (Extended Data Fig. 3d and Supplementary Fig. 7). A comparison of the protein zonation coefficients between the healthy and desmoplastic tissue revealed a general loss of spatial organization. This was evidenced by zonation coefficients shifting closer to zero for both periportal and pericentral zonated proteins, when compared with their corresponding partners in healthy individuals. We quantified this systematic trend by measuring the deviation of the observed regression (solid line) from the expected diagonal of unchanged coefficients (dashed line), with the flatter slope indicating global reduction of zonation magnitude in desmoplasia (Fig. 5a). Notably, while proteins showed reduced zonation, we did not observe any inversion of zonation patterns between conditions. PCA of the desmoplastic samples showed a weaker association between the spatial ratio and the first principle component than observed in the healthy cohort (Spearman’s correlation = −0.31, *P* = 1.7 × 10^−3^; Extended Data Fig. 8a).

To statistically evaluate the proteins driving the reduction in the global zonation profile, we compared the observed difference in zonation coefficients (Δ*β*_1_) with the null hypothesis of zero difference. This analysis identified a subset of proteins (18.1%) with particularly dramatic reductions in their zonation coefficients in desmoplastic tissue, including key metabolic enzymes from both portal vein-associated pathways, such as ASS1, SUCLG2 and AMDHD1, as well as central vein processes, exemplified by CYP2E1, UGT2B7 and ALDH1L1 (Fig. 5b,c). Hence, while disruption of healthy liver architecture globally weakens liver zonation, the effect appears heterogeneous across proteins, with some partly maintaining their spatial organization while others lose it almost entirely. Interestingly, central vein-associated proteins were generally more strongly impacted in their spatial organization than their portal vein counterparts. Notably, these patterns were not driven by any single sample. Although the Individual 17 sample displayed more pronounced tissue alterations, it clustered with other desmoplasia samples and its exclusion yielded consistent results (Supplementary Fig. 8). We further highlighted proteins showing the strongest loss in their zonal expression distribution in the desmoplastic samples by displaying the measured protein expression profiles in Fig. 5d. We validated these findings using immunofluorescence staining as an orthogonal approach. AMDHD1 and UGT2B7 were among the top hits in this analysis, as well as the two known zonation markers ASS1 and GS (Fig. 5e and Extended Data Fig. 8c). These representative immunofluorescence stainings, together with the quantitative analysis, confirmed the loss of spatial organization across all markers (Extended Data Fig. 8d).

**Fig. 6:**
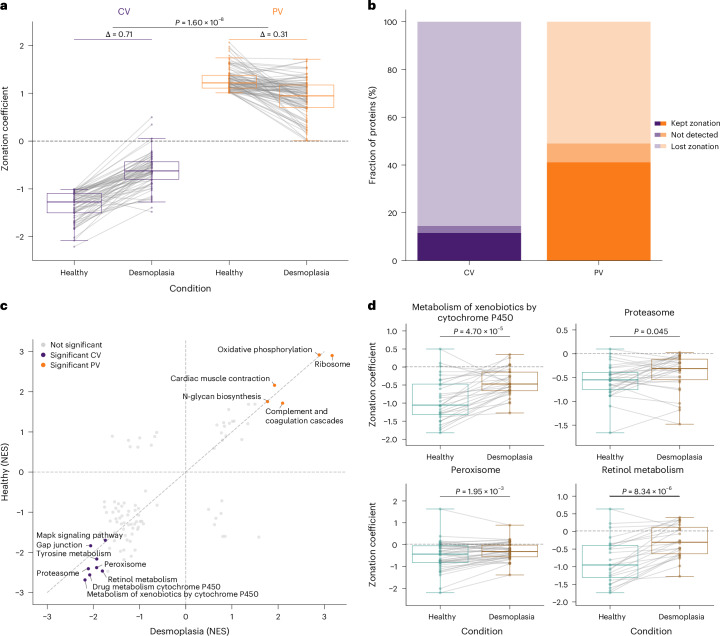
Desmoplasia predominantly affects central vein protein zonation patterns. **a**, Zonation coefficients comparing healthy and desmoplastic tissue. Strongly zonated proteins (|zonation coefficient| > 1 and *Q* < 0.05) from the healthy cohort that are also detected in the desmoplasia cohort are taken into account (central vein: *n* = 67; portal vein: *n* = 94). Grey lines connect matched proteins between conditions. Delta values indicate the median absolute zonation coefficient change between conditions at the respective vein. The *P* value is determined by a two-sided, unpaired rank sum test for the absolute differences between healthy and desmoplasia for each protein. **b**, Fraction of proteins in central and portal vein showing lost (|zonation coefficient| < 1) or maintained (|zonation coefficient| > 1 and *Q* < 0.05) zonation, or not detected in desmoplastic tissue. **c**, Correlation of NES from independent GSEA analysis (KEGG pathways) of the healthy and desmoplasia cohorts, ranked by the zonation coefficient. Pathways significantly enriched (*Q* < 0.1) in both cohorts are colour-coded. **d**, Box plots comparing zonation coefficients between healthy (teal) and desmoplasia (brown) tissue for four selected central vein-enriched KEGG pathways. The *P* values are determined by a two-sided Wilcoxon test for paired samples. Boxes show first and third quartiles, centre line indicates the median, whiskers extend to 1.5× interquartile range, and individual proteins are shown as dots with grey lines connecting matched proteins between conditions. Unless otherwise stated, only proteins at 70% data completeness are included (healthy: *n* = 1,741, *N* = 14; desmoplasia: *n* = 1,684, *N* = 4).

As indicated by the list of proteins with the strongest impact of desmoplasia on their spatial organization, we found that the loss of protein zonation was generally not uniform across the liver lobule. Proteins that were enriched near central vein regions in healthy tissue showed a significantly stronger reduction in their zonation coefficients compared with portal vein proteins, with median zonation coefficient changes of Δ = 0.71 and Δ = 0.31, respectively (Fig. 6a). This discrepancy was also evident at the individual protein levels. Overall, 41.2% of portal vein proteins preserved their zonation in desmoplastic liver tissue, in contrast to only 11.6% of central vein proteins (Fig. 6b).

Similar to the GSEA on zonation coefficients for healthy tissue, we extended this analysis to the desmoplasia cohort (Fig. 3e and Extended Data Fig. 8b). The enriched terms remained consistent between this and the healthy cohort. However, comparing NES values between healthy and desmoplastic tissue for pathways significant in both conditions corroborated the asymmetric change in spatial organization. Central vein-associated metabolic pathways particularly deviated from the diagonal line towards lower enrichment scores in desmoplasia (Fig. 6c), as exemplified by several key metabolic pathways located at the central vein area, including xenobiotic metabolism, proteasome function, peroxisome pathways and retinol metabolism. All of these processes moved closer towards a median zonation coefficient of zero, indicating a general loss of spatial organization in fibrotic tissue (Fig. 6d). Together, these results demonstrate that disruption of physiological liver architecture leads to a pronounced loss in protein zonation profiles, with central vein-specific processes being particularly vulnerable to spatial disorganization.

## Discussion

In this study, we advanced the scDVP framework to enable the analysis of small clinical cohorts, creating an in-depth spatial map of the human hepatic proteome at single-cell resolution. Using a label-free MS approach, we quantified a maximum of nearly 4,000 proteins per individual hepatocyte, achieving a median depth of 2,539 proteins across hundreds of cells (Fig. 2a). To enable this scale-up in cohort size, we developed an automated, strategic cell selection approach to identify cells with optimal distribution along a given spatial trajectory, including automated preservation of spatial metadata. This enabled efficient sampling of cells, maximizing information gain from each tissue section and thereby requiring fewer cells to capture the spatial organization of tissues. The versatility of this selection pipeline extends from fully automated vein identification in healthy tissue to GUI-based manual trajectory definition for complex tissue architectures. We have developed this approach into CellPick (cellpick.app), a comprehensive tool for strategic spatial cell sampling applicable to diverse tissue contexts and biological questions beyond liver zonation.

Furthermore, we created a statistical framework to analyse protein expression changes continuously along spatial trajectories. The human liver is particularly suited to benchmark such an approach, as well-studied metabolic and signalling gradients occur over a relatively short and defined physical distance of approximately 20 hepatocytes or about 385 µm in humans^29^. While earlier liver zonation studies relied on a three-zone model (periportal, mid-lobular, pericentral) or discretized the porto–central axis into equidistant bins, the framework presented here captures the gradual nature of biological processes in the lobule without introducing artificial boundaries^3,17^. This statistical framework should be particularly powerful when optimal numbers of artificial bins are not known a priori. Our approach models protein expression as continuous trajectories through space using an LMM, which accounts for patient-specific variations while avoiding the need to bin the spatial trajectory and reducing sensitivity to missing values, for example, due to dropout samples (Fig. 3a). It generates an easy-to-understand quantitative measure of the degree and directionality of zonation for each protein with increased statistical power compared with binned approaches (Extended Data Fig. 5).

In our study, we confirmed and extended previous findings indicating that half of the hepatic proteome is expressed with spatial variation^10,13,17,28^, among which 171 proteins showed a particularly strong zonation profile. We further characterized the functional zonation created by this extensive shift in the hepatic proteome along the porto–central axis, which uncovered an elegant molecular division of labour that optimizes overall tissue efficiency. This comprehensive and spatially resolved map of the human hepatic proteome is thought to complement existing human transcriptomic datasets, as RNA and protein levels show only moderate spatial correlation^13,30,31^ (Supplementary Fig. 9). It serves as a valuable resource for researchers investigating liver physiology, disease mechanisms and potential therapeutic targets in humans and is accessible to the community through an interactive website (https://human-liver-dvp.streamlit.app/). While demonstrated on liver tissue, our framework, comprising advances in both cell selection and data analysis, is broadly applicable to any spatial proteomics dataset where gradual changes in protein expression occur, such as developmental gradients or tumour microenvironments.

The majority of studies on liver zonation have been conducted in mouse models due to their experimental tractability and the ability to perform controlled manipulations. However, translating findings from mouse studies to human physiology and disease is not always straightforward due to inherent species differences. To address this, we re-analysed our previously published mouse scDVP dataset^17^ using the continuous analysis framework introduced here and compared it with the human dataset (Fig. 4). While a relatively low number of zonated proteins overlapped between species, the spatial organization of core metabolic functions showed high conservation, indicating that liver zonation is remarkably conserved from mice to humans. The results also revealed a pronounced periportal zonation of ribosomal proteins in humans, whereas this pattern was not evident in mice (Fig. 4e,f). The human-specific periportal enrichment may reflect increased protein synthesis demands in oxygen-rich regions, supporting the production of plasma proteins and other secreted factors. While these differences could represent genuine species-specific biology, several biological and methodological covariates may also influence the comparison. Specifically, metabolic state, sex and circadian timing differed between the human and mouse samples, factors known to influence hepatic zonation. A dedicated follow-up study designed to directly evaluate interspecies zonation differences would be needed to clarify the extent and biological significance of these observations. In summary, our comparative approach should assist researchers in identifying species-specific variation and selecting appropriate mouse models to investigate human disease.

To examine the impact of disruptions in liver architecture on zonation, we expanded the analysis to human samples with metastatic liver disease exhibiting desmoplasia. At the proteome level, we found that desmoplasia results in a marked disruption of protein zonation patterns (Fig. 5b). Interestingly, this disproportionately impacted pericentral hepatocytes and related pathways, revealing their vulnerability to spatial disorganization (Fig. 6). The preferential vulnerability of pericentral hepatocytes may relate to the breakdown of lobular gradients, a consequence of the disruptions in liver architecture. Pericentral hepatocytes are particularly dependent on oxygen and Wnt signalling for maintaining their zonated identity and function, potentially explaining their higher vulnerability^6^. Consistent with this, known Wnt target proteins^10^ showed pericentral zonation in healthy tissue and lost this spatial organization in desmoplasia, although whether this reflects disrupted Wnt signalling specifically or the general pattern of pericentral zonation loss remains to be determined (Supplementary Fig. 10). These findings may open interesting avenues for future research to address the role of pericentral hepatocytes in disease progression.

While our approach provides robust spatial analysis capabilities, it operates within certain technical constraints. Our filtering criterion requiring 70% data completeness at the protein level ensures robust statistical analysis across the cohort; however, some highly zonated proteins with restricted expression domains may be excluded from certain analyses due to sparse data coverage (Supplementary Fig. 11). This trade-off between statistical robustness and comprehensive protein coverage is inherent to studies of spatially restricted proteins. Researchers with special interest in these proteins can openly access the unfiltered data via the Proteomics Identifications Database (PRIDE). Moreover, the continuous protein gradient mapping analysis is optimized for linear protein expression gradients. Although nonlinear gradients can still be captured, our model might not optimally fit these patterns. Additionally, the current state-of-the-art MS setup, while robust for single hepatocyte shapes containing about 250 pg of protein (equivalent to one complete HeLa cell), will benefit from further technological advances in sensitivity to expand the application of scDVP to smaller cell types^32^. Lastly, our cross-species comparison is limited by sex imbalance between cohorts, with the human healthy cohort being predominantly female (11 female, 3 male) while the mouse dataset comprised exclusively male animals. This precludes definitive separation of species-specific biological differences from potential sex-based differences in zonation patterns. Future studies with sex-balanced cohorts would be needed to fully resolve this question.

In conclusion, combining automated cell selection and advanced protein gradient mapping creates a powerful framework for spatial proteomics that can be readily adapted to diverse tissue samples and biological questions^15^. Our in-depth spatial hepatic proteome map provides a community resource for understanding human liver organization and may serve as a blueprint for studying protein gradients in other biological systems, from development to disease progression.

## Methods

### Human tissue collection

Human tissue was acquired with written, informed consent under a National Institutes of Health Institutional Review Board-approved protocol (NCT01915225) from two distinct patient populations. Healthy liver samples (*N* = 14) were obtained from patients undergoing risk-reducing total gastrectomy for germline *CDH1* mutation(s), who consented to research liver biopsy during surgery. These patients had hereditary diffuse gastric cancer syndrome but no evidence of liver pathology. Desmoplastic samples (*N* = 4) were obtained from patients with metastatic liver disease undergoing surgical resection, representing regions adjacent to but distinct from metastatic lesions. The tissue procured was characterized by either a normal or tumour–normal margin based upon visible inspection and confirmation of the final histopathologic examination. Following tissue procurement, which was completed roughly within 20 min of incision, tissue was fixed in 1% PFA in PBS for 48 or 72 h, washed in PBS and incubated in 30% sucrose in PBS for at least 24 h before embedding in OCT. OCT blocks were stored at −80 °C. No statistical methods were used to pre-determine sample sizes. The cohort size (18 individuals) represents a substantial increase compared with previous scDVP studies examining spatial trajectories, which analysed up to three mice or six human individuals^17,18^. The number of cells per trajectory (44 hepatocytes) was chosen to provide double coverage of the approximately 20 hepatocytes spanning a human liver lobule, ensuring robust spatial representation. Throughout the paper, uppercase *N* denotes the number of independent biological samples (individuals), while lowercase *n* represents the number of observations (cells, proteins or measurements).

### Histology staining and assessment

Haematoxylin and eosin, Oil Red O and Masson Trichrome histological staining were performed on liver sections for pathological assessment using standard protocols by the Molecular Pathology Unit, National Cancer Institute. Histopathological assessment was performed by a single pathologist and the detailed report can be found in Supplementary Table 4.

### Cryosectioning, tissue mounting and immunofluorescence

Two-micrometre polyethylene naphthalate membrane slides (MicroDissect) were pre-treated by ultraviolet exposure at 254 nm for 60 min to improve tissue adherence. Directly following ultraviolet treatment, sequential washing steps were performed on the slides: first in 350 ml of acetone, then in a solution of 7 ml of VECTABOND reagent (Vector Laboratories; catalogue number SP-1800-7) diluted in 350 ml of acetone, followed by a brief 30-s rinse in ddH_2_O. Slides were then dried under a gentle stream of nitrogen gas. Next, 10-µm-thick liver sections were thawed once and cut onto pre-cooled glass or polyethylene naphthalate membrane slides using a Leica cryostat (Leica CM1950) for histology or DVP purposes, respectively. The slides were stored at −80 °C.

For immunofluorescent staining, the slides were thawed at room temperature followed by a 2-min fixation with 4% PFA in PBS at 37 °C. The tissue was then permeabilized using 95% ethanol in ddH_2_O for 2 min at room temperature, rehydrated with PBS and blocked with 5% BSA in PBS for at least 15 min at room temperature. Following this, sections were incubated with primary antibody GS (abcam; catalogue number ab176562; clone #EPR13022(B); 1:300) overnight at 4 °C. The next day, the sections were washed, three times for 15 min each, with PBS and then incubated with the secondary antibody and fluorescent dyes phalloidin (Thermo Fisher; catalogue number A30104; 1:300) and Sytox Green (Thermo Fisher; catalogue number S7020; 1:500) for 1 h at room temperature. The following day, the sections were washed, three times for 15 min each, with PBS and then incubated at 4 °C overnight with primary antibody ASS1 (abcam; catalogue number ab170952; clone #EPR12398; 1:100) conjugated using Zenon Rabbit IgG Labeling Kits (Thermo Fisher; catalogue number Z25307; 1:20 relative to microlitres of ASS1 used). The next day, after an additional three washes, for 15 min each with PBS, the slides were left unmounted and stored at 4 °C until shipment.

A similar immunofluorescence protocol was used to validate protein distribution. Primary antibodies UGT2B7 (ProteinTech; catalogue number 16661-1-AP; polyclonal; 1:50), AMDHD1 (OriGene; catalogue number TA809954; clone #OTI1D4; 1:250) or RPS3 (abcam; catalogue number ab128995; clone #EPR7808; 1:100) were incubated overnight at 4 °C. The next day, the sections were washed, three times for 15 min each, with PBS and then incubated with secondary antibodies (1:500) and fluorescent dyes DAPI (Thermo Fisher Scientific; catalogue number H3570; 1:1,000) and phalloidin (Thermo Fisher Scientific; catalogue number A22287; 1:300) for 1 h at room temperature. After an additional three washes, for 15 min each with PBS, the slides were mounted in the dark overnight in preparation for imaging.

### High-resolution microscopy, image processing and quantification

For scDVP, confocal image acquisition was performed using a PerkinElmer OperaPhenix high-content imaging system equipped with a ×20 air objective (numerical aperture 0.8). The system was operated through Harmony software v.4.9, with images collected using 2 × 2 binning and 10% overlap between adjacent tiles. For each sample, a single focal plane was manually selected and maintained across all channels. To eliminate channel crosstalk, fluorophores were excited sequentially, with the following acquisition parameters: CFP at 80% laser power and 100-ms exposure, Alexa 488 at 30% power and 20-ms exposure, Alexa 568 at 80% power for 40 ms and Alexa 647 at 60% power for 40 ms. Post-acquisition flat-field correction was applied through the Harmony v.4.9 software platform. Image reconstruction was accomplished using scPortrait^33^, with the phalloidin–CFP signal used as the reference channel for calculating tile positions. These positions were subsequently applied to all channels to generate one tif file per channel per sample. Stitching parameters were set to 0.1 for tile overlap, with filter sigma and maximum shift values of 1 and 50, respectively.

Immunofluorescence images for validation of loss of zonation profiles were acquired using a Leica SP8 inverted confocal laser scanning microscope, a ×63 (1.4 numerical aperture) oil objective, a zoom of 1.00 and a pixel resolution of 1,024 × 1,024. The tile scans were merged using the Leica software and further processed using Imaris (Bitplane 9.9.0) and Image J^34^. Fluorescence intensity ratios or intensities normalized to area were plotted.

### Identification and strategic selection of single cells

The processed and stitched images were imported into the Biological Image Analysis Software (BIAS) using the packaged import tool and retiled to 1,024 × 1,024 pixels with 10% overlap. The region of interest in each image was selected. Cell segmentation was performed externally using a custom-trained Cellpose segmentation model (Cellpose v.2.0) on the phalloidin staining channel^35^. The resulting masks were imported into BIAS where duplicates were removed. Three characteristic reference points were selected based on prominent tissue features per image. Contours and reference points were exported as .xml files. The contours were subsequently simplified by removing at least 90% of the data points defining the contour’s shape.

In this study, only cells belonging to one defined zonation trajectory per patient were analysed. These were selected in an automated fashion: first, portal and central veins were automatically detected in the images using a custom algorithm by which areas with no cells were identified. A Gaussian blur and morphological dilation was applied to the image to remove small voids and subsequently was thresholded using the filters .threshold_minimum function from the skimage package. Central and portal veins were then identified by the expression of the marker proteins GS and ASS1, respectively. All encountered veins were grouped by the algorithm in non-overlapping trajectories, defined by a pair of portal and central veins. The trajectory of interest was selected manually. Defined regions of interest were automatically generated, including all cells segmented directly between the veins and up to an angle of 65 degrees within the axis defined by the centre of both veins (Extended Data Fig. 1). Cells to segment were then chosen such that the minimum distance between any two shapes was maximized by solving a combinatorial optimization problem known as remote-edge diversity maximization using the farthest-first traversal algorithm^19,20^. Up to 44 non-overlapping shapes per pair of central and portal veins were selected to be cut via laser microdissection for further processing. For each of the selected cells, the spatial ratio *S* was calculated, as $S=\frac{{d}_{{\rm{portal}}}}{{d}_{{\rm{portal}}}\,+\,{d}_{{\rm{central}}}}$, with *d*_portal_ the distance (for example, in pixels) from the centre of the shape at hand to the portal vein and *d*_central_ the distance to the central vein. The values from 0 (here close to the central vein) to 1 (here close to the portal vein) are covered uniformly by the selected shapes. To facilitate this analysis workflow, we developed a GUI where users need only to select two points of interest—point A (central vein) and point B (portal vein). An XML file containing the coordinates of the 44 cells along that trajectory is generated automatically by the GUI, enabling straightforward downstream analysis. Generally, only trajectories between clearly identified central and portal veins were analysed. This strategic cell selection approach has since been further developed into CellPick (cellpick.app), a comprehensive web-based tool for flexible spatial sampling in diverse tissue contexts.

### Laser microdissection

Following image alignment using three manually selected tissue reference points (tissue characteristics seen in bright field), cell contours were imported and isolated using a Leica LMD7. The system was operated in a semi-automated manner controlled by the LMD v.8.3 software and using the following optimized parameters: ×63 objective, power of 51–56, aperture 1, speed 10, middle pulse count 1, final pulse 0, offset 100, head current of 50–56% and pulse frequency set to 2,900. The system was aligned to each plate as well as calibrated to account for gravitational stage shift when collecting into low-binding 384-well plates (Eppendorf; catalogue number 0030129547). Single cells were sorted into every other well and the outer rows and columns were left empty. Visual confirmation of successful cutting was performed using the live imaging interface. To minimize air currents and maximize collection efficiency, the entire LMD system was operated in a sealed incubator environment. A protective shield plate was positioned above the sample stage to avoid collection errors. The collection plates were sealed, centrifuged at 1,000*g* for 2 min and frozen at −20 °C until further processing. Collection success was assessed post acquisition through data-driven quality control based on protein identification numbers, yielding an 82% effective collection rate (649 of 792 samples; see ‘Data filtering and quality control’).

### Sample preparation and peptide loading

The following sample preparation steps were performed on an Agilent Bravo automated liquid handling platform in a semi-automated manner to minimize sample loss. Additionally, the plates were sealed with PCR sealing foil during incubation steps. Plates containing single cells were removed from −20 °C and immediately centrifuged at 2,000*g* for 2 min. The wells were washed with 28 μl of 100% ACN and subsequent dried in a vacuum concentrator at 45 °C for 20 min to ensure the presence of cut shapes at the bottom of the well. Then, 6 μl of lysis buffer (0.013% DDM in 60 mM TEAB buffer, pH 8.5) was added per well. Lysis was performed at 95 °C for 60 min in a PCR cycler (lid temperature 110 °C). We added 1 μl of 80% ACN and the samples were cooked at 75 °C for an additional 60 min. After a brief cooling, 1 μl of digestion mixture containing 4 ng μl^−1^ LysC and 6 ng μl^−1^ trypsin in 60 mM TEAB buffer was added and incubated overnight (approximately 18 h) at 37 °C. On the next day, the samples were immediately frozen at −20 °C until sample loading was performed.

The samples were loaded on C-18 tips (Evotip Pure, Evosep). The plates were thawed, immediately centrifuged at 2,000*g* for 2 min and kept on ice until loading. Evotips were activated in 1-propanol for 3 min. The tips were washed twice with 50 μl of buffer B (0.1% formic acid, 99.9% ACN) and centrifuged at 700*g* for 1 min between washes. A second activation in 1-propanol was performed for 3 min, followed by two washing steps with 50 μl of buffer A (0.1% formic acid). The disk was kept wet after the final wash. Then, 70 μl of buffer A was added per tip and the samples were loaded. Each well was rinsed with 10 μl of buffer A and added to the respective tip. For the peptide binding step, centrifugation was performed at 700*g* for 2 min. The tips were washed with 50 μl of buffer A. Finally, 150 μl of buffer A was added as overlay and the tips centrifuged at 700*g* for 15 s. The loaded tips were stored for maximum 3 days in an Evotip box containing fresh buffer A until loading on the LC-MS system.

### LC–MS data acquisition

MS acquisition order was randomized across samples within each Evotip box. Analysis was performed on an Evosep One liquid chromatography system (Evosep) coupled to an Orbitrap Astral mass spectrometer (Thermo Fisher Scientific). An EASY-Spray source (Thermo Fisher Scientific) operating at 1,900 V connected the two systems. Peptide separation was achieved using a Whisper Zoom 80 SPD gradient on an Aurora Rapid C18 column (5 cm, 75-μm internal diameter, 1.7-μm particle size, IonOptics) at 60 °C. A throughput of 80 samples per day was achieved.

The Orbitrap Astral was equipped with a FAIMS Pro interface (−40-V compensation voltage, 3.5 l min^−1^ carrier gas, Thermo Fisher Scientific) and sample acquisition was exclusively performed in DIA mode. Orbitrap MS1 scans were recorded at a resolution of 240,000, a scan range from 380 to 980 *m*/*z* using 500% normalized AGC target and 100-ms maximum injection time. The window width of the Astral MS/MS scans was optimized in accordance to the sample’s precursor density. A total of 60 variable-width isolation windows were defined which covered the precursor selection range of 380 to 980 *m*/*z* (Supplementary Table 2). Fragment ion spectra were acquired with 20-ms maximum injection time, 500% normalized AGC target and 25% higher-energy collisional dissociation (HCD) collision energy.

### Raw data processing

All 792 raw files were analysed in one single library-free search using DIA-NN (v.1.8.1)^36^. A human reference proteome FASTA file from UniProt (UP000005640, including reviewed entries, downloaded 22 March 2024) was provided. The search was performed with match-between-runs enabled and mass accuracy set to 8 and MS1 accuracy to 4 and the scan window radius to 6. Protein inference was based on genes using heuristic protein grouping. A maximum of one missed cleavage was allowed. The output ‘report.tsv’ file was normalized using directLFQ (v.0.2.19) at standard settings^37^. The resulting ‘report.tsv.protein_intensities.tsv’ file was used for downstream data analysis steps. No data imputation was applied.

### Bioinformatics data analysis

Data collection and analysis were not performed blind to the conditions of the experiments. Non-parametric tests were used where applicable. For *t*-tests, data distribution was assumed to be normal but was not formally tested.

#### Data filtering and quality control

Initial data processing was performed using Python (v.3.12.5). At the sample level, measurements were excluded where the number of identified proteins was either 1.5 s.d. below or 3 s.d. above the median number of identifications across all samples. Quality control analyses, including assessment of protein group distributions and protein intensity rank plots, were performed on this filtered dataset to verify data quality and consistency. For subsequent analyses, an additional filtering criterion at the protein level was applied, requiring proteins to be detected in at least 70% of samples, if not stated otherwise. Where indicated for downstream analyses, data were standardized on the protein level using *Z*-score normalization within each patient. By this, protein intensities are centred around the mean and are scaled to unit variance on a per-patient basis.

#### Continuous analysis approach

To explore protein expression gradients along the zonation trajectories for each patient, an LMM was fitted, using the spatial score (*S*) as an independent variable, and protein expression (*y*) as the corresponding dependent outcome^27^. A random intercept effect on the patient the cell came from was added to control for differing expression baselines in different samples. Cells were removed by outlier intensity values using Tukey’s fences method^38^. The resulting expression intensities were normalized using a robust scaler (by which the median is removed and the interquartile range is divided). The full model is then given by following equation:

$${y}_{{ij}}={\beta }_{0}+{\beta }_{1}{S}_{{ij}}+{u}_{i}+{\epsilon }_{{ij}}$$

where *y*_*ij*_ is the response variable for cell *j* in patient *i*, *β*_0_ is the fixed intercept, *β*_1_ is the fixed slope, from here on called zonation coefficient, for the predictor variable *S*_*ij*_, *u*_*i*_ is the random intercept for patient *i*, where ${u}_{i}\approx {{{\mathbb{N}}}}{\mathbb{(}}0,{\sigma }_{u}^{2})$, and *ε*_*ij*_ is the residual error term, where ${\epsilon }_{{ij}}\approx {{{\mathbb{N}}}}{\mathbb{(}}0,{\sigma }_{u}^{2})$.

Once fitted, these models can be used to explore significant spatial expression gradients, by testing the zonation coefficient *β*_1_ against the null hypothesis that it equals zero (expression is spatially uniform in the central–portal axis) via a Wald test. The estimated zonation coefficients are compared with their standard error to determine the likelihood that the observed effect could occur by chance. A protein expression gradient that increases from portal to central vein is indicated by a zonation coefficient significantly lower than zero. In contrast, an expression gradient that increases towards the portal vein is indicated by a significant positive zonation coefficient. The strength of the gradient is determined by the absolute value of the zonation coefficients (*β*_1_). Proteins with a zonation coefficient greater than 1 ((∣*β*_1_∣ > 1) and a *Q* value less than 0.05 were considered significantly zonated.

To evaluate whether protein zonation gradients differ between experimental conditions, the zonation coefficients (*β*_1_) of the LMMs fitted for controls were compared with those of individuals with desmoplastic liver tissue. Only proteins with significant zonation gradients in the control condition were selected, and their zonation coefficients were compared using a Wald test. The null hypothesis that the difference in zonation coefficients (Δ*β*_1_) between the two conditions is zero is assessed by the Wald test. A test statistic that is distributed normally under the null hypothesis is obtained by dividing the observed difference by its standard error.

#### Statistical analysis and data visualization

PCA was performed using scikit-learn after data standardization using StandardScaler, by which each feature is centred and scaled to unit variance. Before PCA, outliers were removed using a *Z*-score-based approach, where samples with any feature having an absolute *Z*-score greater than 3 were excluded from further analysis. Explained variance ratios were calculated for each component. Cell type contamination analysis was performed to quantitatively validate hepatocyte purity using a cell-type-resolved liver proteome dataset from ref. ^25^. Cell-type-specific proteins were defined as proteins with intensity higher than 1 × 10^8^ and at least fivefold higher than in any other cell type. The top 20 proteins with highest intensity were selected from each cell-type-specific protein set, compiling marker lists for human hepatocytes, hepatic stellate cells, Kupffer cells and liver endothelial cells. For each individual isolated cell, median ranks were calculated for each cell-type-specific marker protein set to assess potential contamination, with undetected proteins assigned the lowest rank of zero. For spatial analysis, both continuous and discrete approaches were employed. The continuous analysis is explained in detailed below. For discrete analysis, data were divided into 20 equidistant bins along the porto–central axis, and mean protein expression was calculated for each bin. *Z*-scores were calculated using scipy.stats.zscore function along the bin axis, with NaN values omitted from the calculation. LOWESS smoothing (fraction = 0.4) was applied to protein intensities plotted against the porto–central ratio of selected proteins to visualize their spatial behaviour. Moreover, protein expression heatmaps were used for visualization and proteins were ordered by the absolute expression difference along the spatial trajectory. Hierarchical clustering was performed using Ward’s method with Euclidean distance to identify general protein expression patterns. Overrepresentation analysis was performed using Enrichr’s web interface with the complete dataset as background. GSEA was performed using MSigDB collections (KEGG Pathways and MSigDB Hallmark) with proteins ranked by zonation coefficients from the LMM. Statistical significance was assessed using Benjamini–Hochberg correction for multiple testing. For visualization of entire pathways, the median *Z*-scores of the proteins assigned to the respective pathway were calculated. For comparison between analysis approaches on the protein level, protein intensities were analysed using both continuous (LMM-based) and discrete methods. For the discrete analysis, protein intensities were tested for spatial differences using one-way ANOVA implemented through ordinary least squares regression with statsmodels. Expression differences between the 20 spatial bins were tested using Type II sum of squares. *P* values were adjusted for multiple testing using the Benjamini–Hochberg procedure to control the false discovery rate. The statistical significance (−log_10_(*Q* value)) from both approaches was compared, and correlation between methods was assessed using Spearman correlation. A significance threshold of *Q* < 0.05 was applied for both methods. Moreover, kernel density estimation with 20 density levels was used to visualize the distribution of significance values. The comparison on the pathway level was conducted using both 3-bin and 20-bin approaches. GSEA was performed analogue as described above with proteins ranked by *F*-statistics from the respective ANOVA analyses. Statistical comparisons between conditions were performed using two-sided Wilcoxon tests for paired samples and two-sided unpaired rank sum tests for absolute differences. Delta values for zonation coefficient changes were calculated as the median absolute difference between conditions. Regression analyses included 95% confidence interval calculations for trajectory visualization. First and third quartiles are shown by box plots with centre line indicating median and whiskers extending to 1.5× interquartile range. All visualizations were generated using seaborn and matplotlib libraries in Python.

#### Transcriptomic–proteomic correlation

We used the MERFISH spatial transcriptomics dataset from ref. ^11^ to identify landmark genes that are highly expressed in either pericentral or periportal hepatocytes. We defined landmark genes as those expressed in at least 70% of cells in a given region (central or portal) and showing a differential expression score of at least 30 between the two zones. The selected landmark genes were then used to computationally infer zonation^39^. Using this landmark-based approach, we next computed zonation scores on a single-nucleus RNA sequencing dataset from healthy human hepatocytes^40^. Once zonation scores were established for each cell, we applied a linear mixed-effects model to calculate zonation coefficients, mirroring the approach used for our proteomics data. To ensure consistency with the proteomics normalization and noise reduction, we included only RNA with a median expression greater than zero across healthy hepatocytes. Finally, we compared the RNA zonation coefficients with their matched proteins (*n* = 419 RNA–protein pairs).

#### Cross-species comparison analysis

Cross-species conservation of protein zonation was assessed by comparison of human liver data with the publicly available mouse scDVP dataset^17^. Protein homology mapping between human and mouse was performed using DIOPT (DRSC Integrative Ortholog Prediction Tool), by which multiple orthology prediction algorithms are integrated to provide comprehensive homology scores^41^. The protein filtering and normalization strategies described in the original paper were maintained. The mouse data were then re-analysed using our continuous analysis approach to enable direct comparison of zonation patterns between species.

### Reporting summary

Further information on research design is available in the Nature Portfolio Reporting Summary linked to this article.

## Supplementary information

Supplementary Information Supplementary Figs. 1–11.

Reporting Summary

Supplementary Tables Supplementary Table 1. Clinical characteristics of study cohort. Supplementary Table 2. Variable window scheme for data-independent acquisition (DIA). Supplementary Table 3. Spatial proteome expression results. Supplementary Table 4. Histopathological assessment of surgical liver samples.

## Source data

Source Data Extended Data Fig. 8 Immunofluorescence image quantification values (Extended Data Fig. 8d).

## Data Availability

The mass spectrometry proteomics data have been deposited to the ProteomeXchange Consortium via the PRIDE partner repository with the dataset identifier PXD062231. Furthermore, the analysed data are openly accessible at an easy-to-use interactive website (https://human-liver-dvp.streamlit.app/). Imaging data have been deposited to BioImages^42^ with the accession number S-BIAD2529. Source data are provided with this paper.

## Code availability

All custom code developed for this study is publicly available from GitHub. The cell selection pipeline, including automated vein detection and the graphical user interface for manual trajectory definition, is available via https://github.com/BorgwardtLab/Human_scDVP_CellSelection. The analysis code for sample filtering and continuous zonation analysis, along with the processed data files (directLFQ output and filtered data frames), is available via https://github.com/BorgwardtLab/Human_scDVP_Analysis. The code underlying the interactive data visualization application is available via https://github.com/BorgwardtLab/Human_scDVP_data_dashboard. Additionally, the cell selection methodology has been further developed into CellPick, a standalone application for strategic spatial cell sampling, available via https://github.com/BorgwardtLab/CellPick.
