# Supplementary material for: Single-cell spatial proteomics maps human liver zonation patterns and their vulnerability to disruption in tissue architecture
Source: Nat Metab. 2026 Feb 20;8(3):741–56. doi: 10.1038/s42255-026-01459-2 (PMC13031132; doi:10.1038/s42255-026-01459-2)
Supplement: Supplementary file 1 — Supplementary Figs. 1–11. [file 42255_2026_1459_MOESM1_ESM.pdf]

nature metabolism

Supplementary information https://doi.org/10.1038/s42255-026-01459-2

Single-cell spatial proteomics maps human liver zonation patterns and their vulnerability to disruption in tissue architecture

In the format provided by the authors and unedited

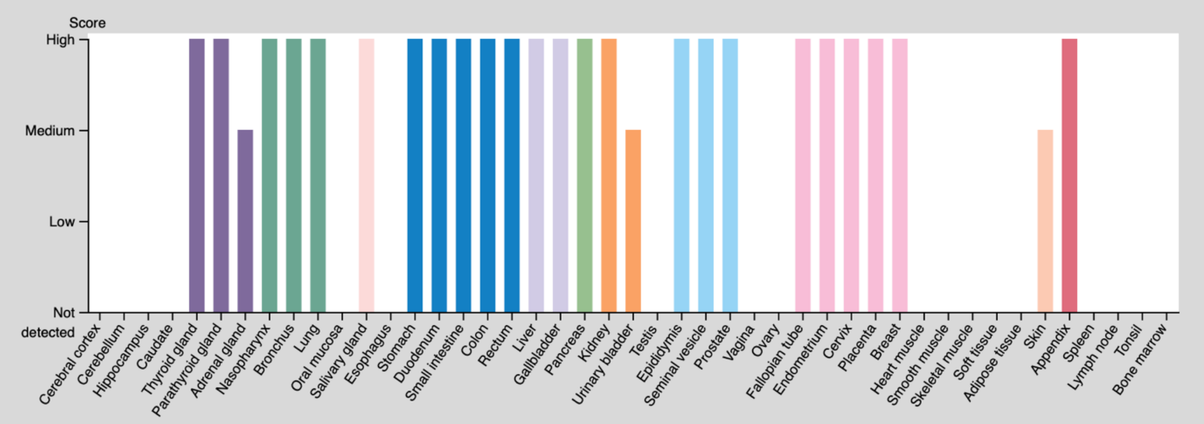

Fig. S1, KRT18 occurrence in human tissues. The graph is extracted from the Human Protein Atlas tissue section (https://www.proteinatlas.org/ENSG00000111057-KRT18/tissue - 30.09.2025). Protein expression in human tissues is assessed by immunohistochemistry and reported with the units not detected, low, medium and high.

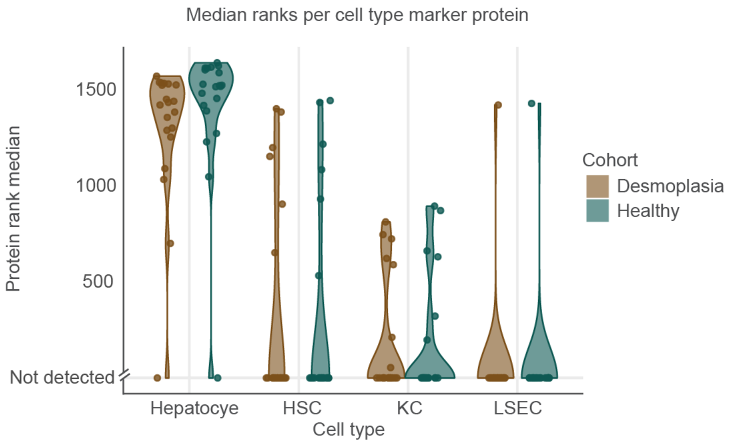

Fig. S2, Cell type-specificity analysis confirms hepatocyte purity. Violin plots showing median protein ranks for cell type-specific marker proteins across conditions. Each dot represents one marker protein for the respective cell type (n = 20 proteins per cell type).

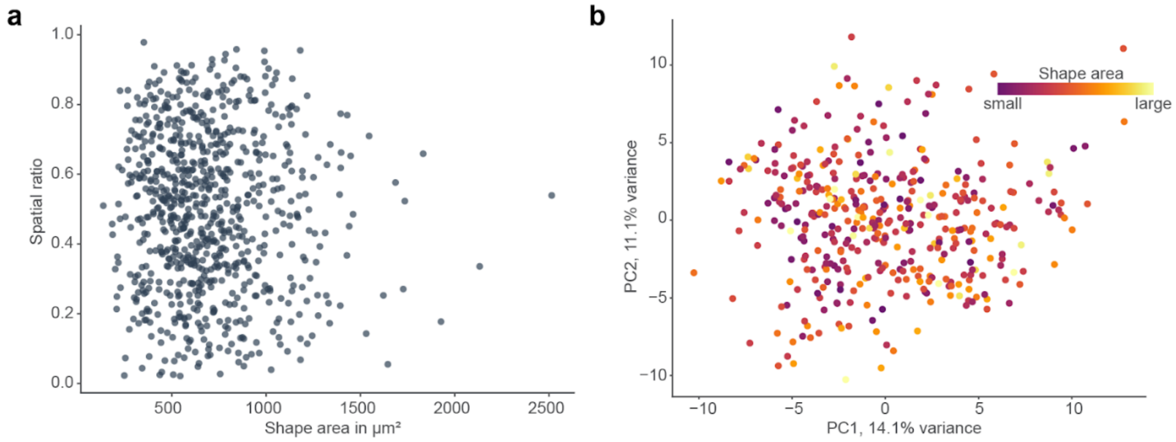

Fig. S3, Shape area does not correlate with spatial position in tissue sections. a) Scatter plot showing the relationship between isolated shape area (µm²) and spatial ratio S across all analyzed cells. No significant correlation was observed (Spearman's correlation = -0.042). **b)** Principal component analysis colored by shape area demonstrates that cell size does not drive the main variance in the proteome data, with PC1 primarily reflecting spatial zonation rather than morphometric differences.

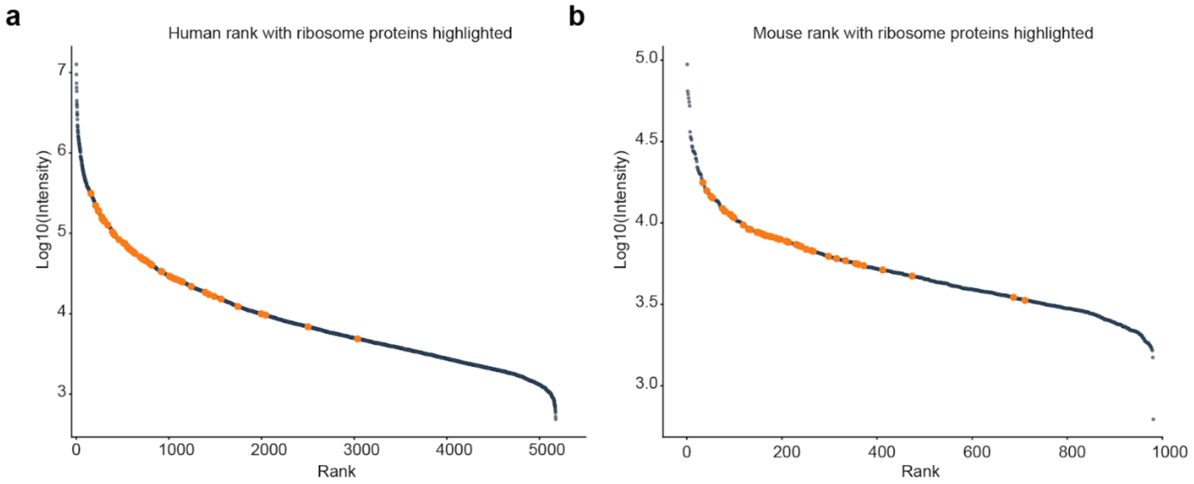

Fig. S4, Ribosomal proteins are highly abundant in both human and mouse datasets. Protein abundance rank plots showing log10 intensity versus rank for **a)** human and **b)** mouse scDVP datasets. Ribosomal proteins (highlighted in orange) are among the most abundant proteins in both species.

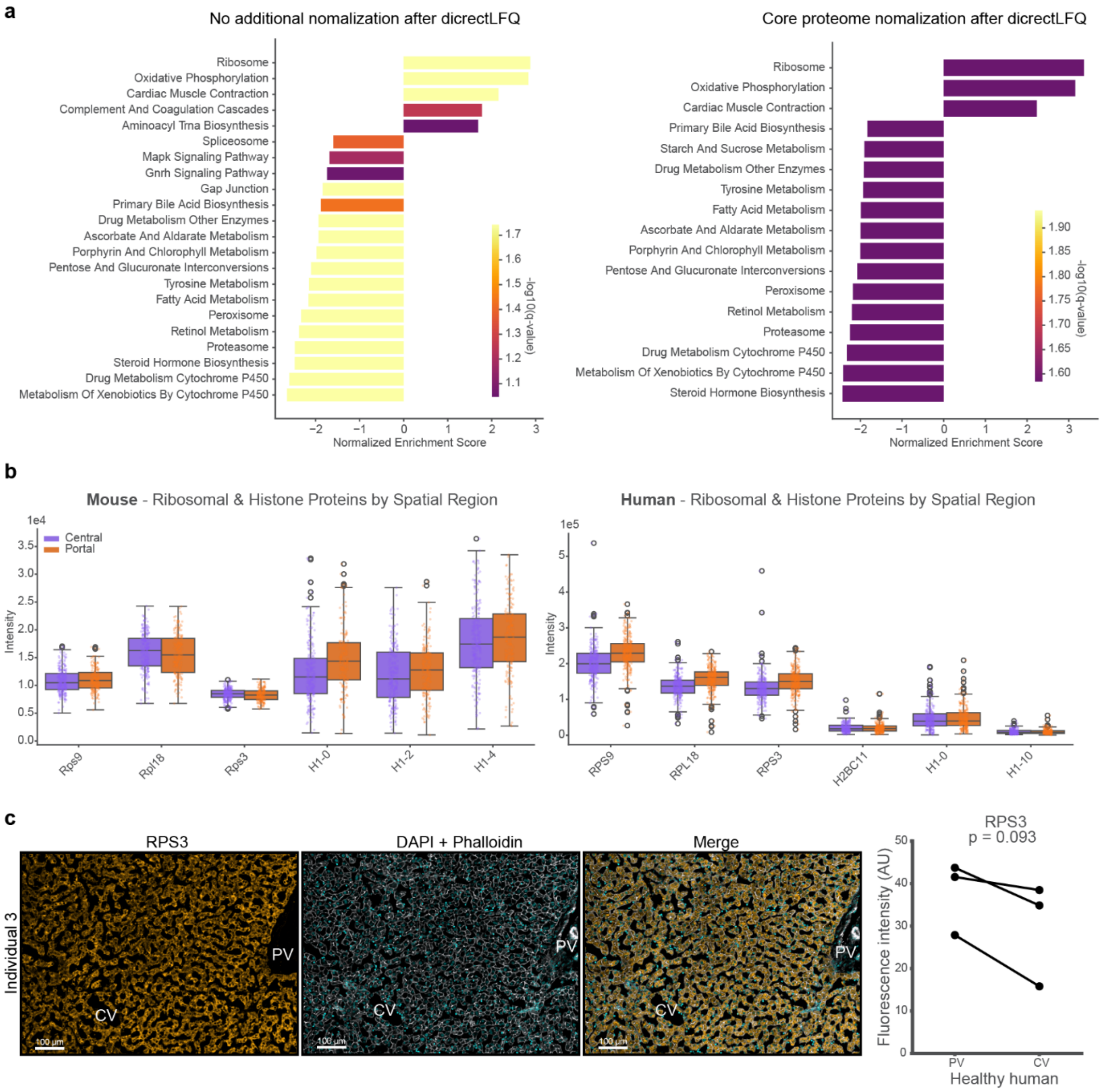

Fig. S5, Ribosomal protein analysis using alternative normalization methods and immunofluorescence staining. a) Gene set enrichment analysis (GSEA) of healthy human liver dataset using KEGG pathway database with two normalization approaches. Left: directLFQ normalization as used in the main analysis (**Fig. 3e**). Right: directLFQ followed by core proteome normalization. Proteins were ranked by zonation coefficient in both analyses. Normalized enrichment scores (NES) are shown with color gradient indicating $-\log_{10}$(q-value). Statistical significance assessed using Benjamini-Hochberg correction for multiple testing. **b)** Box plots showing protein intensities for selected ribosomal (RPS9, RPL18, RPS3) and histone (H2BC11, H1-0, H1-10) proteins in central (purple, spatial ratio $S \leq 0.5$) and portal (orange, spatial ratio $S > 0.5$) regions. Boxes show first and third quartiles, center line indicates median, whiskers extend to 1.5× interquartile range. Individual hepatocyte measurements are shown as dots overlaid on boxplots. Left: Mouse (N=3), Right: Human (N=14) **c)** Immunofluorescence staining of ribosomal protein RPS3 in healthy human liver tissue (Individual 3). Left: RPS3 channel (orange). Middle: DAPI + phalloidin nuclear staining (cyan, white). Right: Merged image. Fluorescence intensity was quantified per region across three individuals (healthy human) and compared using a two-sided paired t-test ($p = 0.093$, N = 3). PV: portal vein, CV: central vein. Scale bars, 100 µm.

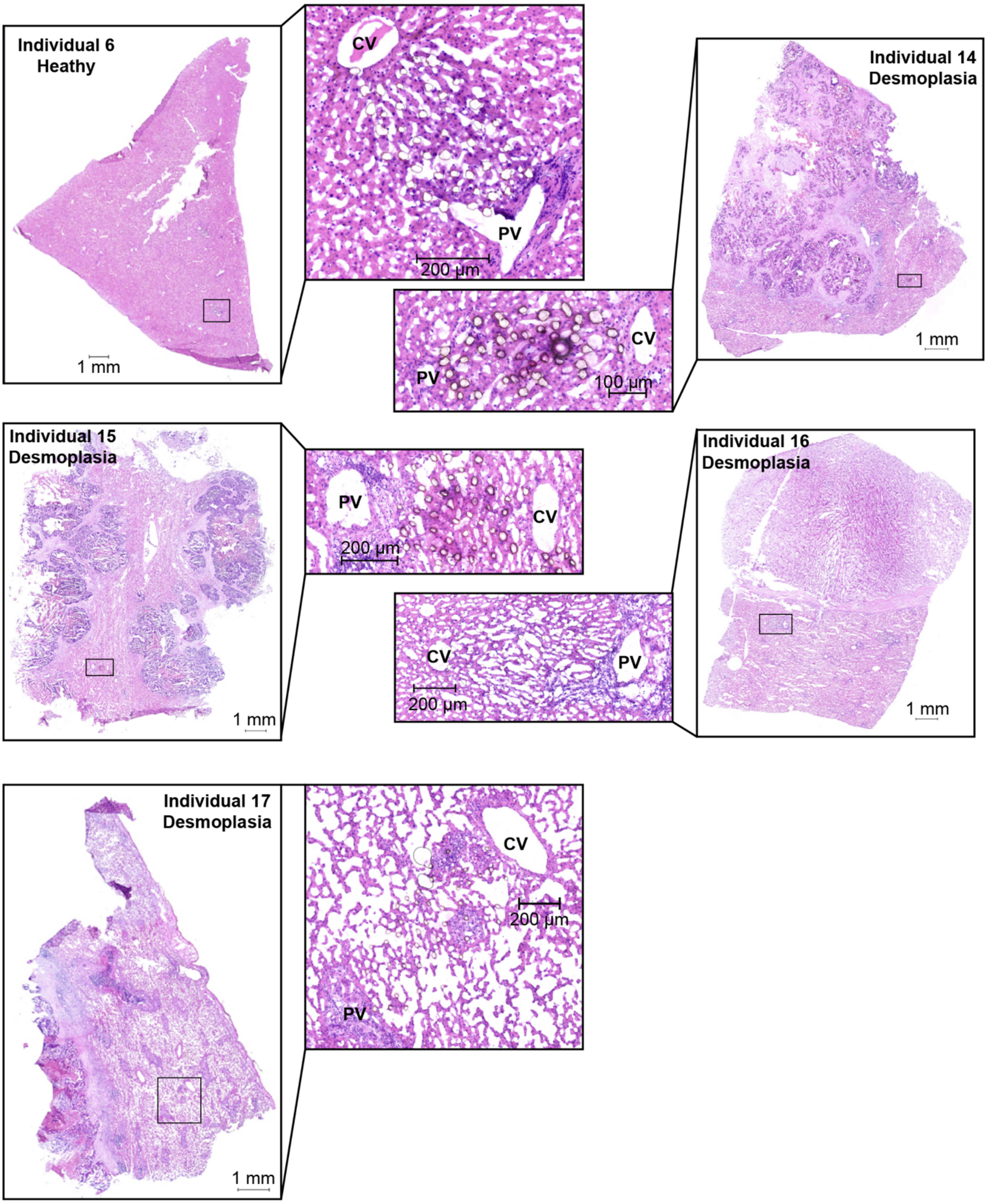

Fig. S6, H&E staining of tissue sections used for laser microdissection. H&E staining was performed on the same tissue sections used for four-channel DVP staining and laser microdissection. The overview images indicated by black boxes the location of the analyzed trajectories. Images were acquired after hepatocytes were laser-dissected for downstream proteomics analysis. Zoomed regions are labelled with portal veins (PV) and central veins (CV) and display holes left by laser-cut cells. One representative healthy individual (Individual 6) and all four desmoplasia individuals (Individuals 14, 15, 16, and 17) are shown. Scale bars are as indicated.

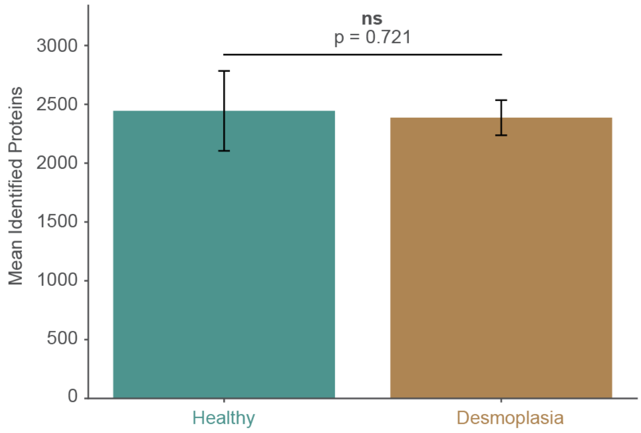

Fig. S7, Comparable protein identification depth across healthy and desmoplasia samples. Group-level comparison showing mean protein identification numbers with standard deviation error bars for healthy versus desmoplasia cohorts. Statistical significance determined by Mann-Whitney U test ($U = 32.0$, $p = 0.721$, $N_H = 14$, $N_D = 4$).

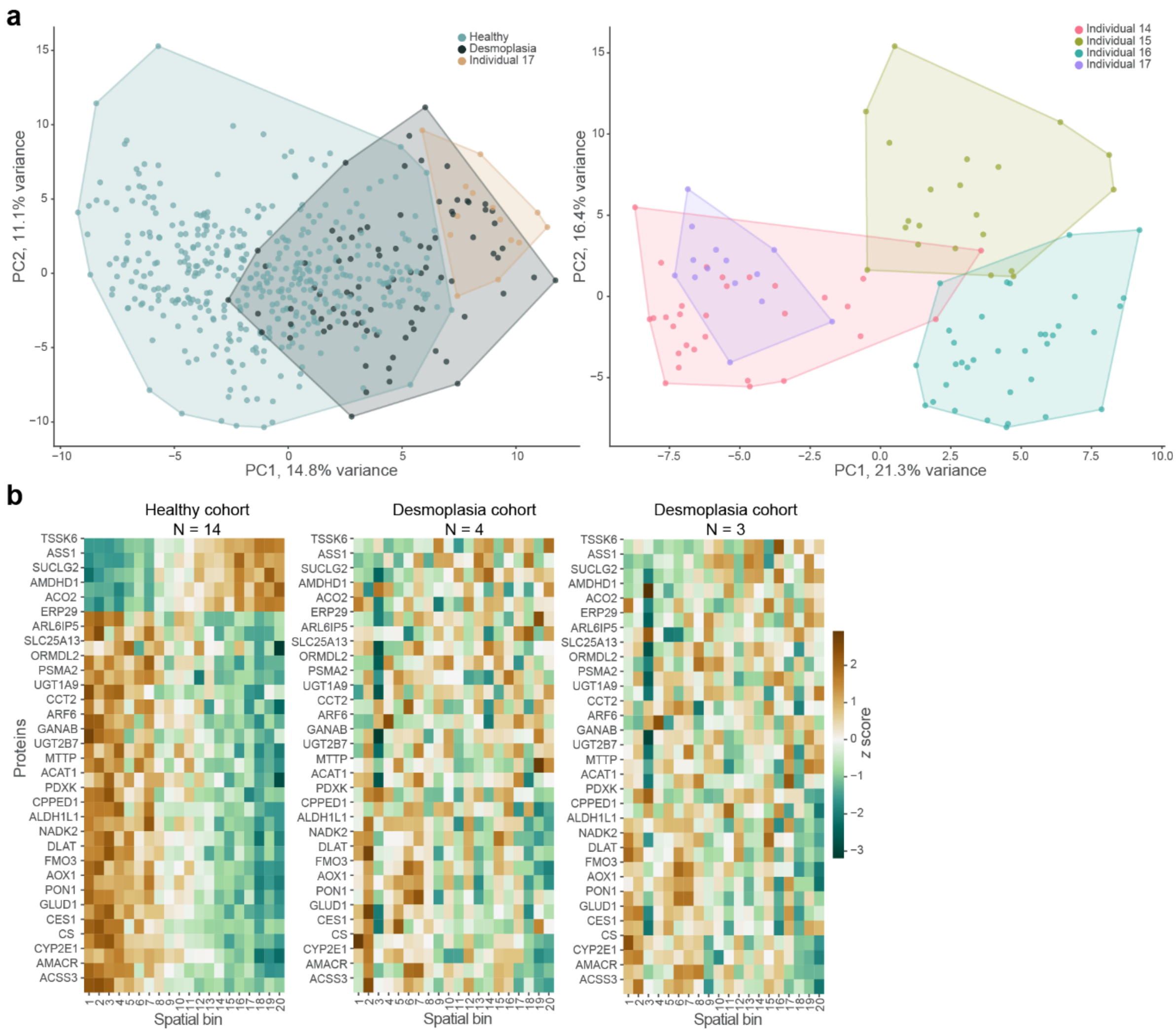

Fig. S8, Individual 17 shows consistent behavior with other desmoplasia samples. a) Left: Principal component analysis (PCA) of single-cell proteomes from the entire cohort after outlier removal, with samples colored by cohort (healthy: teal, desmoplasia: grey, individual 17: orange; n = 513, N = 18). Right: PCA of desmoplasia cohort alone, with samples colored by individual (n = 100, N = 4). **b)** Protein expression heatmaps (z scored) of proteins showing significant zonation loss in desmoplasia (identified in **Fig. 5b**) across 20 equal-width spatial bins from central (spatial ratio S = 0) to portal (spatial ratio S = 1). Left: healthy cohort (N = 14), middle: desmoplasia cohort including all four individuals (N = 4), right: desmoplasia cohort excluding individual 17 (N = 3).

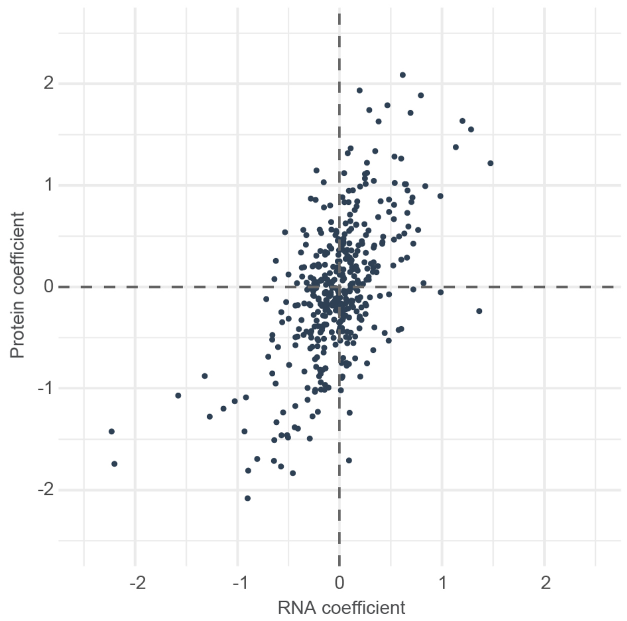

Fig. S9, Comparison of zonation coefficients between transcriptomic and proteomic data. Scatter plot comparing zonation coefficients derived from single-nucleus RNA sequencing data (x-axis) versus single-cell proteomics data (y-axis) for the intersection of the datasets (n = 419). Each dot represents one transcript-protein pair. Dashed lines indicate zero zonation coefficient for both modalities.

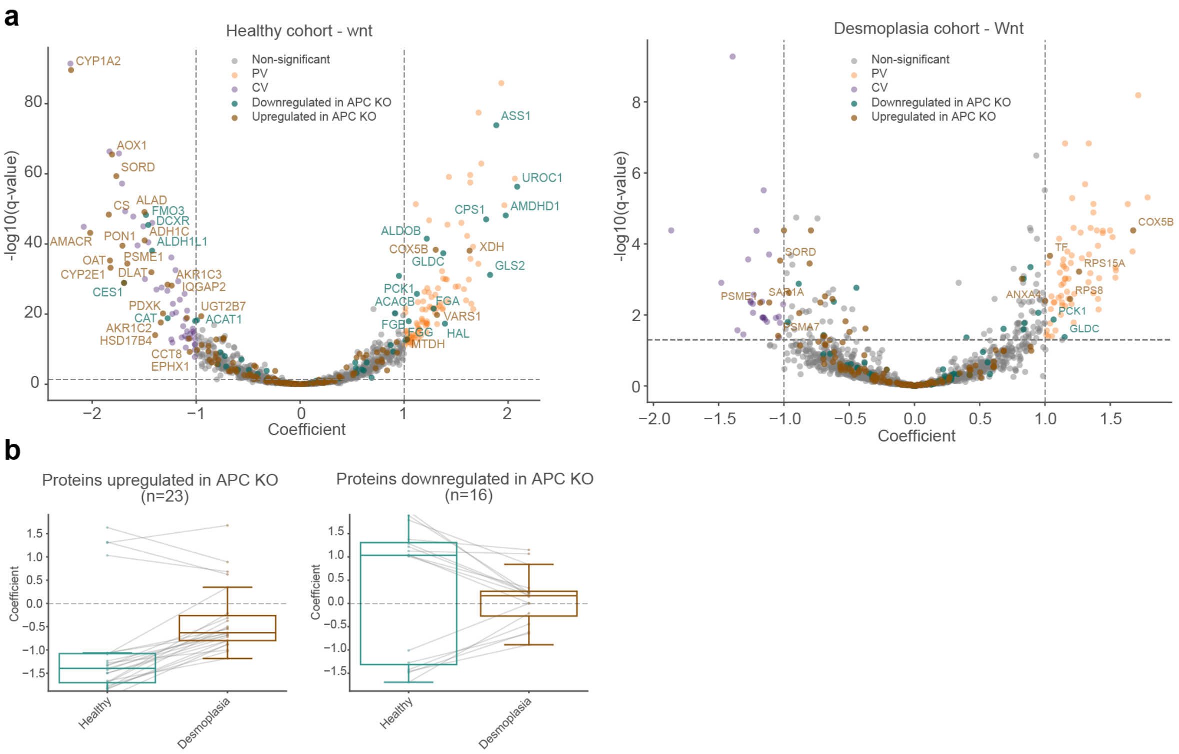

Fig. S10, Illustration of Wnt target protein zonation in healthy and desmoplastic tissue. a) Volcano plots showing results of the continuous analysis for healthy (left) and desmoplasia (right) cohorts. Each protein zonation coefficient determined by linear mixed modeling is plotted against the -log10(q-value) from a Wald test. Wnt target proteins are color-coded based on their APC fold change values: proteins upregulated in APC KO are shown in brown, while downregulated proteins are shown in teal. Proteins previously reported as strongly zonated towards the PV and CV are shown in orange and purple, respectively. Gray dots represent non-significant proteins. **b)** Box plots below compare zonation coefficients between healthy (teal) and desmoplasia (brown) tissue for Wnt target proteins upregulated (left, n=23) and downregulated upon APC KO (right, n=16). Boxes show first and third quartiles, center line indicates the median, whiskers extend to 1.5x interquartile range, and individual proteins are shown as dots with gray lines connecting matched proteins between conditions.

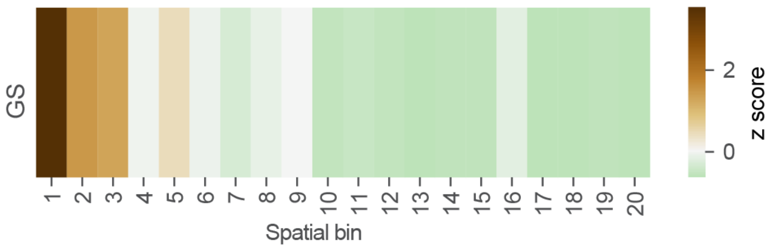

Fig. S11, Spatial expression pattern of glutamine synthetase (GS) across liver zonation. Heatmap showing z-scored expression of GS across 20 equal-width spatial bins from the central vein (bin 1) to the portal vein (bin 20).
