# Supplementary material for: Single-cell spatial proteomics maps human liver zonation patterns and their vulnerability to disruption in tissue architecture
Source: Nat Metab. 2026 Feb 20;8(3):741–56. doi: 10.1038/s42255-026-01459-2 (PMC13031132; doi:10.1038/s42255-026-01459-2)
Supplement: Supplementary file 2 — Reporting Summary [file 42255_2026_1459_MOESM2_ESM.pdf]

nature portfolio

Corresponding author(s): Matthias Mann

Last updated by author(s): Sep 29, 2025

Reporting Summary

Nature Portfolio wishes to improve the reproducibility of the work that we publish. This form provides structure for consistency and transparency in reporting. For further information on Nature Portfolio policies, see our Editorial Policies and the Editorial Policy Checklist.

Statistics

For all statistical analyses, confirm that the following items are present in the figure legend, table legend, main text, or Methods section.

n/a	Confirmed	
☐	☒	The exact sample size (*n*) for each experimental group/condition, given as a discrete number and unit of measurement
☐	☒	A statement on whether measurements were taken from distinct samples or whether the same sample was measured repeatedly
☐	☒	The statistical test(s) used AND whether they are one- or two-sided<br>*Only common tests should be described solely by name; describe more complex techniques in the Methods section.*
☐	☒	A description of all covariates tested
☐	☒	A description of any assumptions or corrections, such as tests of normality and adjustment for multiple comparisons
☐	☒	A full description of the statistical parameters including central tendency (e.g. means) or other basic estimates (e.g. regression coefficient) AND variation (e.g. standard deviation) or associated estimates of uncertainty (e.g. confidence intervals)
☐	☒	For null hypothesis testing, the test statistic (e.g. *F*, *t*, *r*) with confidence intervals, effect sizes, degrees of freedom and *P* value noted<br>*Give P values as exact values whenever suitable.*
☒	☐	For Bayesian analysis, information on the choice of priors and Markov chain Monte Carlo settings
☒	☐	For hierarchical and complex designs, identification of the appropriate level for tests and full reporting of outcomes
☐	☒	Estimates of effect sizes (e.g. Cohen's *d*, Pearson's *r*), indicating how they were calculated

Our web collection on statistics for biologists contains articles on many of the points above.

Software and code

Policy information about availability of computer code

Data collection	PerkinElmer Harmony v4.9, Single-Cell Technologies BIAS (September 2023), scPortrait, HALO v3.6.4134.362, Leica LMD version 8.3, Orbitrap Astral Tune Application 1.0.100.40, Cellpose 2.0, python v3.11, numpy v2.2.3, pandas v2.2.3, seaborn v0.13.2, statsmodels v.0.14.4, scikit-learn v1.6.1
Data analysis	DIA-NN v1.8.1, , directLFQ v0.2.19, python v3.11, decoupler v1.8.0, matplotlib v3.8.2, matplotlib-venn v1.1., missingno v0.5.2, mygene v3.2.2, numpy v1.26.3, pandas v2.2.0, scikit-image v0.22.0, scikit-learn v1.4.0, scikit-learn-extra v0.3.0, scipy v1.12.0, seaborn v0.13.2, statsmodels v0.14.1, tqdm 4.66.1, pillow v10.2.0, untangle v1.2.1, lxml v5.1.0, joblib v1.3.2, snakemake v.7.32.4, opencv-ptyhon v4.9.0.80.<br>All custom code developed for this study is publicly available on GitHub. The cell selection pipeline, including automated vein detection and the graphical user interface for manual trajectory definition, is available at https://github.com/BorgwardtLab/Human_scDVP_CellSelection. The analysis code for sample filtering and continuous zonation analysis, along with the processed data files (directLFQ output and filtered data frames), is available at https://github.com/BorgwardtLab/Human_scDVP_Analysis. The code underlying the interactive data visualization application is available at https://github.com/BorgwardtLab/Human_scDVP_data_dashboard. Additionally, the cell selection methodology has been further developed into CellPick, a standalone application for strategic spatial cell sampling, available at https://github.com/BorgwardtLab/CellPick.

For manuscripts utilizing custom algorithms or software that are central to the research but not yet described in published literature, software must be made available to editors and reviewers. We strongly encourage code deposition in a community repository (e.g. GitHub). See the Nature Portfolio guidelines for submitting code & software for further information.

Data

Policy information about availability of data

All manuscripts must include a data availability statement. This statement should provide the following information, where applicable:

- Accession codes, unique identifiers, or web links for publicly available datasets
- A description of any restrictions on data availability
- For clinical datasets or third party data, please ensure that the statement adheres to our policy

The mass spectrometry proteomics data have been deposited to the ProteomeXchange Consortium via the PRIDE partner repository with the dataset identifier PXD062231. Futhermore, the analyzed data is openly accessible at an easy-to-use interactive website (https://human-liver-dvp.streamlit.app/). Imaging data has been deposited to BioImages with the accession number S-BIAD2529.

Research involving human participants, their data, or biological material

Policy information about studies with human participants or human data. See also policy information about sex, gender (identity/presentation), and sexual orientation and race, ethnicity and racism.

Reporting on sex and gender	Clinical metadata including sex are summarized in Supplementary Data Table 1. Both male and female samples were included without prior selection due to the small cohort size.
Reporting on race, ethnicity, or other socially relevant groupings	Socially constructed categorization was not taken into consideration. Patient material was sampled at the National Institute of Health, Bethesda, MD, USA.
Population characteristics	Clinical metadata including age, gender and BMI are summarised in Extended Data Table 1. Individual samples were explicitly de-identified to comply with European and country-specific General Data Protection Regulation (GDPR).
Recruitment	Participants were recruited from patients undergoing surgery at the National Institutes of Health under protocol NCT01915225. Healthy liver samples were obtained from patients undergoing risk-reducing gastrectomy for germline CDH1 mutations; desmoplasia samples from patients undergoing resection for metastatic liver disease. Cohort composition was determined by sample availability, resulting in predominantly female participants (13F/5M). CDH1 mutations in the healthy cohort do not affect liver pathology. These factors are unlikely to impact the primary findings on liver zonation patterns.
Ethics oversight	Human tissue was acquired with informed consent under an NIH IRB-approved protocol (NCT01915225) for surgical resection or risk-reducing surgery performed on patients with germline CDH1 mutation(s).

Note that full information on the approval of the study protocol must also be provided in the manuscript.

Field-specific reporting

Please select the one below that is the best fit for your research. If you are not sure, read the appropriate sections before making your selection.

☒ Life sciences ☐ Behavioural & social sciences ☐ Ecological, evolutionary & environmental sciences

For a reference copy of the document with all sections, see nature.com/documents/nr-reporting-summary-flat.pdf

Life sciences study design

All studies must disclose on these points even when the disclosure is negative.

Sample size	N = 18 individuals (14 healthy, 4 desmoplasia). No statistical methods were used to pre-determine sample sizes. Cohort size was oriented on and increased compared to previous scDVP studies (refs 17, 18). 44 cells per trajectory were chosen to provide double coverage of the ~20 hepatocytes spanning a human liver lobule.
Data exclusions	At the sample level, measurements were excluded where the number of identified proteins was either 1.5 standard deviations below or 3 standard deviations above the median number of identifications across all samples. Quality control analyses, including assessment of protein group distributions and protein intensity rank plots, were performed on this filtered dataset to verify data quality and consistency. In principle component analysis (PCA), additional outlier removal based on z-scores was applied for clearer visualization (exclusion: samples with any feature having a z-score greater than 3 standard deviations. For subsequent analyses, an additional filtering criterion at the protein level was applied, requiring proteins to be detected in at least 70% of samples, if not stated otherwise.
Replication	44 cells per patient per trajectory were measured. Only one technical replica per single cell was measured. Reproducibility was ensured through biological replication across 18 individuals (14 healthy, 4 desmoplasia). Consistent zonation patterns were observed across healthy individuals and loss of zonation in desmoplasia was confirmed by orthogonal IF validation (N=3 per condition).
Randomization	Patient samples were processed in random order. MS acquisition was randomized within batches of 3 patients and MS performance was tightly monitored with HeLa QC runs before, during, and after each experimental block.

Blinding

Cell selection was automated and driven by a farthest-first traversal algorithm without human input. Investigators were not blinded to experimental conditions as samples were analyzed by group (healthy vs. desmoplasia).

Reporting for specific materials, systems and methods

We require information from authors about some types of materials, experimental systems and methods used in many studies. Here, indicate whether each material, system or method listed is relevant to your study. If you are not sure if a list item applies to your research, read the appropriate section before selecting a response.

Materials & experimental systems

n/a	Involved in the study
☐	☒ Antibodies
☒	☐ Eukaryotic cell lines
☒	☐ Palaeontology and archaeology
☒	☐ Animals and other organisms
☒	☐ Clinical data
☒	☐ Dual use research of concern
☒	☐ Plants

Methods

n/a	Involved in the study
☒	☐ ChIP-seq
☒	☐ Flow cytometry
☒	☐ MRI-based neuroimaging

Antibodies

Antibodies used

Glutamine Synthetase (GS; abcam; ab176562; clone# EPR13022(B); 1:300), ASS1 (abcam; ab170952; clone# EPR12398; 1:100), UGT2B7 (ProteinTech; 16661-1-AP; polyclonal; 1:50), AMDHD1 (OriGene; TA809954; clone# OTI1D4; 1:250), RPS3 (abcam; ab128995; #EPR7808; 1:100)

Validation

All antibodies are commercially available and were validated by the manufactures' standard procedure. ab176562 (GS): KO validation and advanced validation; ab170952 (ASS1): KO validated; 16661-1-AP (UGT2B7): validated for Immunofluorescence using HepG2 cells; TA809954 (AMDHD1) validated for Western Blot in HEK293T cells and immunohistochemistry in paraffin-embedded human liver tissue; ab128995 (RPS3): validated for Western Blot in HepG2, PC-12, and Neuro-2a cells; immunohistochemistry in paraffin-embedded human kidney tissue; and immunofluorescence in Neuro-2a cells.

Plants

Seed stocks

Report on the source of all seed stocks or other plant material used. If applicable, state the seed stock centre and catalogue number. If plant specimens were collected from the field, describe the collection location, date and sampling procedures.

Novel plant genotypes

Describe the methods by which all novel plant genotypes were produced. This includes those generated by transgenic approaches, gene editing, chemical/radiation-based mutagenesis and hybridization. For transgenic lines, describe the transformation method, the number of independent lines analyzed and the generation upon which experiments were performed. For gene-edited lines, describe the editor used, the endogenous sequence targeted for editing, the targeting guide RNA sequence (if applicable) and how the editor was applied.

Authentication

Describe any authentication procedures for each seed stock used or novel genotype generated. Describe any experiments used to assess the effect of a mutation and, where applicable, how potential secondary effects (e.g. second site T-DNA insertions, mosiacism, off-target gene editing) were examined.
